# Effective Identification of Bacterial Type III Secretion Signals Using Joint Element Features

**DOI:** 10.1371/journal.pone.0059754

**Published:** 2013-04-04

**Authors:** Yejun Wang, Ming’an Sun, Hongxia Bao, Qing Zhang, Dianjing Guo

**Affiliations:** 1 School of Life Sciences and the State Key Lab of Agrobiotechnology, the Chinese University of Hong Kong, Shatin, N.T., Hong Kong; 2 Genome Research Center, Harbin Medical University, Harbin, China; Purdue University, United States of America

## Abstract

Type III secretion system (T3SS) plays important roles in bacteria and host cell interactions by specifically translocating type III effectors into the cytoplasm of the host cells. The N-terminal amino acid sequences of the bacterial type III effectors determine their specific secretion via type III secretion conduits. It is still unclear as to how the N-terminal sequences guide this specificity. In this work, the amino acid composition, secondary structure, and solvent accessibility in the N-termini of type III and non-type III secreted proteins were compared and contrasted. A high-efficacy mathematical model based on these joint features was developed to distinguish the type III proteins from the non-type III ones. The results indicate that secondary structure and solvent accessibility may make important contribution to the specific recognition of type III secretion signals. Analysis also showed that the joint feature of the N-terminal 6^th^–10^th^ amino acids are especially important for guiding specific type III secretion. Furthermore, a genome-wide screening was performed to predict *Salmonella* type III secreted proteins, and 8 new candidates were experimentally validated. Interestingly, type III secretion signals were also predicted in gram-positive bacteria and yeasts. Experimental validation showed that two candidates from yeast can indeed be secreted through *Salmonella* type III secretion conduit. This research provides the first line of direct evidence that secondary structure and solvent accessibility contain important features for guiding specific type III secretion. The new software based on these joint features ensures a high accuracy (general cross-validation sensitivity of ∼96% at a specificity of ∼98%) in silico identification of new type III secreted proteins, which may facilitate our understanding about the specificity of type III secretion and the evolution of type III secreted proteins.

## Introduction

Bacteria encode different protein translocation systems, via which various bacterial substrate proteins are translocated into the host cells in order to function in pathogenesis or symbiosis [1]–[3]. Type III secretion system (T3SS) is particularly important because it mediates and maintains bacterial infection in a wide range of gram-negative bacteria [1]–[3]. Many severe infectious diseases are closely related with T3SSs, including human (and/or animal) plague, typhoid, dysentery, cholera and enteritis, plant blast and streak disease, etc [1], [3]. T3SSs also play important roles in the symbiosis process between *Rhizobia* or other gram-negative symbiotic bacteria and their hosts [1], [3].

The substrates translocated through T3SSs are T3S (Type III Secreted) effectors, which can be specifically recognized and secreted through the T3SS conduit [4]–[5]. After entering the host cell cytoplasm, these effectors can interact with the host proteins and mediate bacterial infection or invasion. Due to their importance in bacteria-host interaction, identification of new T3S effectors has attracted much research attention in the past decade. However, possibly due to bacterial adaptation to different hosts or environments, the number of T3S effectors varies greatly among different bacterial species, and the sequences lack apparent similarity among different effectors [1]–[3]. This makes it extremely difficult to identify new T3S effectors by sequence alignment or phylogenetic approaches. Other features were therefore used to identify new T3S effectors. For example, based on the fact that some effector-coding genes are clustered with T3SS apparatus-encoding genes in a single operon or genomic region [3], [6]–[9], new T3S effectors were identified [7], [10]–[13]. In addition, other general properties, such as distinct G+C nucleotide content, clustering with chaperones, transcriptional co-regulation with apparatus genes, etc., were also used for screening new effectors that scatter in the genomes [14]–[16].

With the tremendous progress of sequencing technology, more and more bacterial genomes have been sequenced [3]. The research interest gradually shifts from individual discovery of effectors to genome-wide identification of effector coding genes [17]–[18]. Two foundational discoveries greatly accelerated the computational identification of new T3S effectors. One is that the N-terminal peptide sequences of T3S effectors contain both necessary and sufficient signal information to guide the specific protein secretion, although there is the argument that T3S signals are encoded in the mRNA but not the amino acid sequences of the T3S effectors [19]–[20]. However, experimental evidence show that some effectors are unable to secrete through T3SS conduit without N-terminal peptides, and the N-terminal peptides can mediate type 3 secretion of some non-effectors [19]–[20]. By computational modeling, Arnold *et al.* and Wang *et al.* found that the frame-shift of T3S signal sequences has more influence on T3S recognition compared to the amino acid position shift [17], [21]. Wang *et al.* further discovered that the T3S effectors which can tolerate frame shift in fact retain their original amino acid composition after the frame shift. This further demonstrates that the N-terminal peptide sequences of T3S effectors indeed encode the T3S signal [21]. The other foundational discovery is that a T3S effector can be secreted through different T3SS conduits [22]–[23]. Based on these discoveries, new features including the N-terminal signal sequence patterns, amino acid composition frequency, and secondary structure composition, etc. were analyzed for T3S proteins [17]–[18], [21], [24]–[26]. The most important features identified so far are sequence-based or position-based amino acid composition (Aac) profiles in the N-terminal signal region [17], [21]. However, the amino acid preference in the signal sequences is quite subtle and the enriched or depleted amino acids do not contain apparent physical and chemical properties. Therefore, no common motif or simple linear amino acid combination has been disclosed from the signal peptide of T3S effectors [26]. Several computational methods were developed to train these atypical features, but unfortunately they only achieved limited success [17]–[18], [21], [24]–[26].

To interpret the possible connections between the subtle but unique Aac features and the specificity of protein secretion, several research groups analyzed the second-order structure composition encoded by the primary signal peptide sequences, including the secondary structure (Sse) and water accessibility states (Acc) [17], [21], [24]. Although distinctive Sse and Acc features were noted, it seems that these features do not individually contribute to the specific recognition of T3S proteins [17], [21], [24]. One group considered the joint distribution of Sse or Acc and Aac, and provided limited evidence that the Sse and Acc features contribute to the specific secretion of T3S proteins [25]. The exact mechanism underlying specific recognition and secretion of T3S proteins are still poorly understood.

In this study, we further explore the possible contribution of secondary structure and solvent accessibility to the specific T3S recognition. We developed a joint-feature distribution model to integrate position-specific Aac, Sse and Acc features of the T3S signal sequences. The model, namely T3SEpre, achieves a high sensitivity of 95.9% at a specificity of 97.7% (5-fold cross validation). The model is robust, inter-species effective, and outperforms the other current software with the same application. An in silico deletion analysis identified the most important region for type III signals. Furthermore, genome-wide T3S prediction was conducted for *Salmonella* and selected predictions were validated experimentally. Interestingly, T3S signals were also identified from gram-positive bacteria and yeasts. Some candidates from yeast were further validated experimentally.

## Results

### 1. Distinct Structural Features of T3S N-terminal Sequences

A comprehensive list of validated T3S effectors were annotated from different bacteria, followed by two-rounds of filtering process to remove homologs for full-length proteins and N-terminal 100aa signal segments (Methods and Materials). The sequences of N-terminal 100aa were extracted for analysis because previous study indicated that this region contain T3S guiding signals [21]. The resulting non-redundant and reliable dataset was subjected to position-specific Aac, Sse and Acc profile analysis.

**Figure 1:**
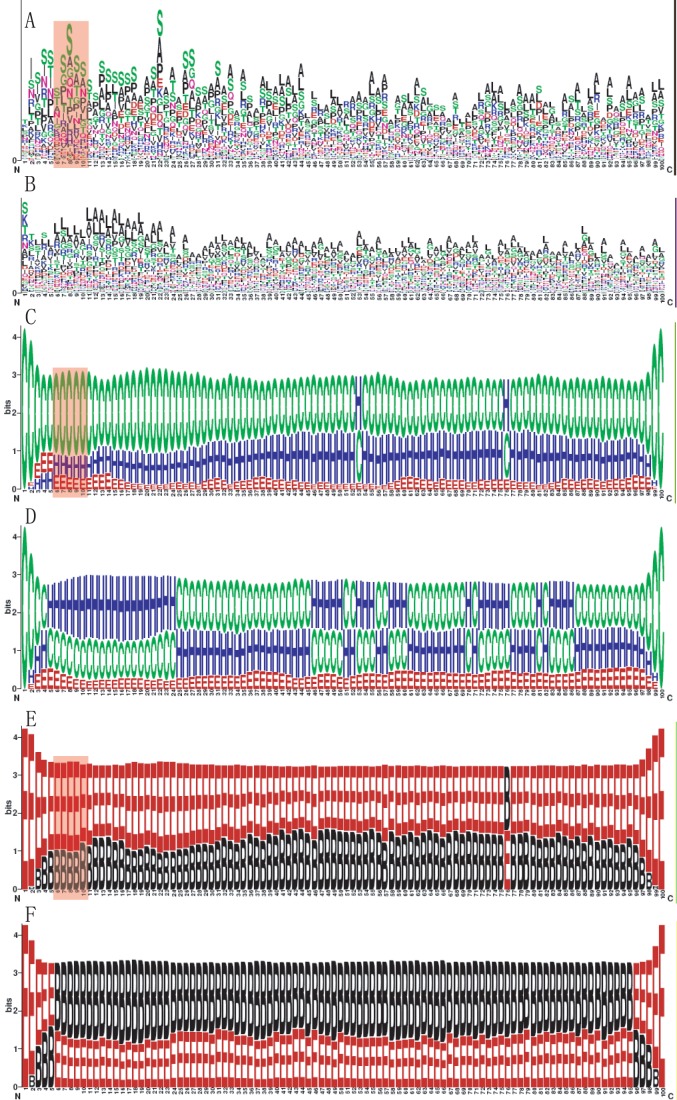
Distinctive N-terminal position-specific Aac, Sse and Acc feature in T3S proteins. Element positions are depicted on the horizontal axis. The heights of characters represent the preference or enrichment level. (**A**), (**C**) and (**E**): Aac, Sse and Acc preference for T3S proteins, respectively. (**B**), (**D**) and (**F**): Aac, Sse and Acc preference for non-T3S proteins, respectively.

Consistent with previous observations [21], serine is apparently enriched in the T3S sequences compared with non-T3S proteins (Fig. 1A and 1B). Secondary structure comparison revealed apparently enriched coils for most positions in the T3S signal sequences (Fig. 1C). This pattern is especially apparent within the first 30 positions (Fig. 1C). In contrast, helices are more preferred at ∼25 positions of the non-T3S sequences (Fig. 1D). In addition, fewer strands are adopted for T3S sequences (Fig. 1C and 1D). Solvent accessibility analysis showed that most positions are exposed for T3S sequences but buried for non-T3S sequences (Fig. 1E and 1F). Taken together, apart from specific Aac features, T3S sequences also contain distinctive Sse and Acc profiles. More coils and fewer strands in the T3S signal regions indicate the sequences may be more flexible [27].

### 2. Distinct Joint Profiles of Sse, Acc and Aac in T3S Signal Sequences

Previous studies suggested that individual Sse or Acc features almost make no contribution to the specific recognition of T3S proteins [17], [21]. In these studies, however, the authors assumed that the Sse and Acc variables were independent of Aac. Alternatively, we consider Sse, Acc and Aac as co-variables depending on each other, and the joint profiles of these 3 features were observed for each position of signal sequences of T3S and non-T3S proteins.

**Figure 2:**
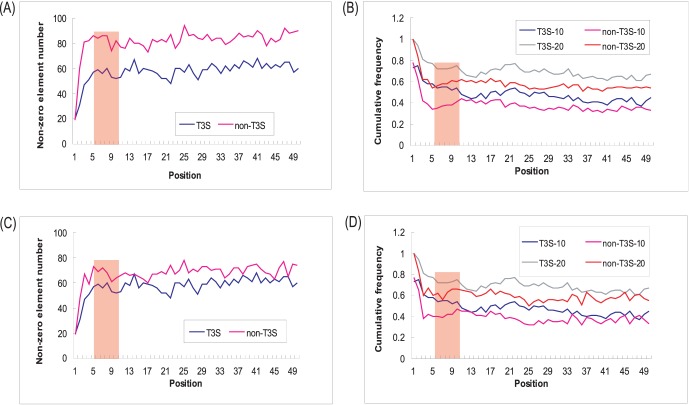
Comparison of preference profile for Aac-Sse-Acc joint features between T3S and non-T3S sequences. (A) and (C): Total number of non-zero distributed joint features at each position for T3S or non-T3S sequences. Full set of joint features include 120 different elements. The ratio of data size between T3S and non-T3S proteins is ∼1∶2 in (A) and 1∶1 in (C). (B) and (D): Cumulative frequency of the most enriched 10 (T3S-10 or non-T3S-10) or 20 (T3S-20 or non-T3S-20) joint features in T3S or non-T3S sequences. The ratio of data size between T3S and non-T3S proteins was about 1∶2 in (B) and 1∶1 in (D). Only the first 50 positions at the N-terminal end of T3S and non-T3S sequences were included for analysis.

As shown in Fig. 2A, T3S proteins exhibit more apparent joint element preference than non-T3S proteins. Specifically, there are apparently fewer elements present in each position of T3S N-terminal sequences. For most positions, the cumulative occurrence frequency for the top 10 and top 20 elements are both higher for T3S proteins (Fig. 2B). ‘SCe’ (‘serine-coil-exposed’) is most frequently preferred by T3S proteins for most positions, followed by ‘TCe’ (‘threonine-coil-exposed’), ‘PCe’ (‘proline-coil-exposed’), ‘NCe’ (‘asparagine-coil-exposed’), ‘GCe’ (‘glycine-coil-exposed’), etc. (Table S1). The difference is still striking when the number of non-T3S and T3S is equal (Fig. 2C and 2D), indicating the general joint element preference in T3S proteins is not caused by smaller data size. Non-T3S proteins also show preference for certain elements, especially within the first 25 positions, and yet the preferred elements are apparently different. For example, ‘LHb’ (‘leucine-helix-buried’), ‘AHb’ (‘alanine-helix-buried’), and ‘VHb’ (‘valine-helix-buried’) are more frequently found in the non-T3S proteins (Table S1).

### 3. T3S Protein Prediction Model Based on Joint Features of Aac, Sse and Acc

The position-specific joint element features were extracted using Bi-profile Bayes (BPB) model [28], and then trained with Support Vector Machine (SVM). The parameters were optimized and shown in Table 1. The new classifier, namely T3SEpre, achieved excellent classifying performance, with a sensitivity of 95.9% at a high specificity of 97.7% (Table 1) in a 5-fold cross-validation.

**Table 1:** Optimal parameters and corresponding performance based on five-fold cross-validation.

Name	C|γ^a^	*Sn* (%) vs. *Sp* (%)	*A* (%)	AUC (%)	MCC
T3SEpre	4|0.001	95.9% vs. 97.7%	97.1	99.5	0.935
BPBAac	8|0.001	84.4% vs. 94.8%	91.3	96.4	0.803
BPBAll	8|0.001	82.0% vs. 95.2%	91.1	96.0	0.796
SSE-ACC	4|0.008	78.0% vs. 95.2%	89.5	94.5	0.759

a *C*: cost, which was optimized based on 10-fold cross-validation grid search.

γ: gamma, which was optimized based on 10-fold cross-validation grid search.

The T3SEpre, BPBAac and BPBAll used BPB model while SSE-ACC used SPB model to extract features from N-terminal 100 amino acids of T3S proteins. All software adopted SVM kernel radial basis function.

**Figure 3:**
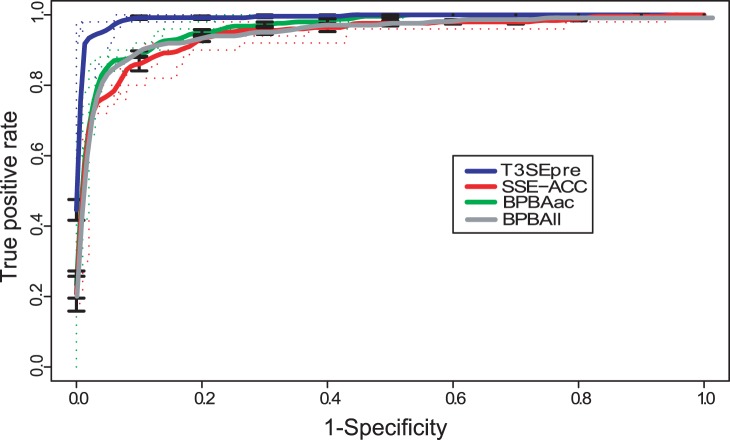
Performance evaluation of T3SEpre. ROC curves resulted from different T3S protein prediction software based on 5-fold cross validation using the same datasets. The parameters were optimized respectively (refer to Table 1).

We found that the Sse and Acc feature made important contribution to the specificity of T3S signals. BPBAac, which adopts the position-specific Aac feature only, is one of the best T3S protein classification programs [21]. A direct comparison showed that T3SEpre outperformed BPBAac with the same training dataset (Fig. 3; Table 1). A BPBAll model was also trained with the current datasets based on the simple linear combination of Aac, Sse and Acc features [21]. Consistent with previous results, the discriminative performance of BPBAac was slightly better than BPBAll [21]. This indicates that Sse and Acc feature do not independently contribute to the T3S specificity, rather in an Aac-dependent manner (Fig. 3; Table 1). Furthermore, T3SEpre was compared with SSE-ACC, a T3S classifier using SVM to train sequence-based but not position-specific features. As shown in Fig. 3 and Table 1, T3SEpre also outperformed SSE-ACC in terms of sensitivity, specificity, accuracy, MCC and AUC of ROC curve. Therefore, the position-based features are proved to be more effective in distinguishing T3S proteins.

To make a thorough comparison, independent datasets were also tested. First, two large-scale T3S protein datasets, Arnold 2009 [17] and Lower 2009 [18], were used. Arnold 2009 contains 109 high-quality validated T3S effectors from different species [17]. Lower 2009 contains 533 partially validated T3S effectors [18], [21]. For both datasets, T3SEpre performed apparently better than BPBAac, especially in terms of sensitivity, accuracy, and MCC values (Table 2). T3SEpre also outperformed earlier software Effective T3 (Table 2). In addition, other two new datasets (Mukaihara 2010 and Baltrus 2011) containing validated T3S effectors from an individual bacterial species or genus [29], [30] were also adopted. Mukaihara 2010 contains a group of validated *Ralstonia* T3S effectors while Baltrus 2011 is a comprehensive set of validated *Pseudomonas* T3S effectors [29], [30]. For Mukaihara 2010, T3SEpre correctly recalled 32 out of the total 35 non-homologous effectors (91.4%), whereas BPBAac and Effective T3 only recalled ∼60% of them (Table 2). T3SEpre also recalled much more known Baltrus 2011 effectors (Table 2).

**Table 2:** Performance comparison using different datasets.

Dataset	Software	*Sn* (%)	*Sp* (%)	*A* (%)	MCC
Lower2009	T3SEpre	59.0	96.2	79.9	0.627
	BPBAac	38.6	99.4	72.8	0.505
	Effective T3	39.4	98.0	72.3	0.495
Arnold2009	T3SEpre	92.7	94.5	93.9	0.869
	BPBAac	86.4	97.7	93.9	0.865
	Effective T3	55.5	94.5	81.4	0.595
Mukaihara2010	T3SEpre	91.4 (32/35)	–	–	–
	BPBAac	60.0 (21/35)	–	–	–
	Effective T3	57.1 (20/35)	–	–	–
Baltrus2011	T3SEpre	83.2 (242/291)	–	–	–
	BPBAac	49.5 (144/291)	–	–	–
	Effective T3	58.1 (169/291)	–	–	–

**Figure 4:**
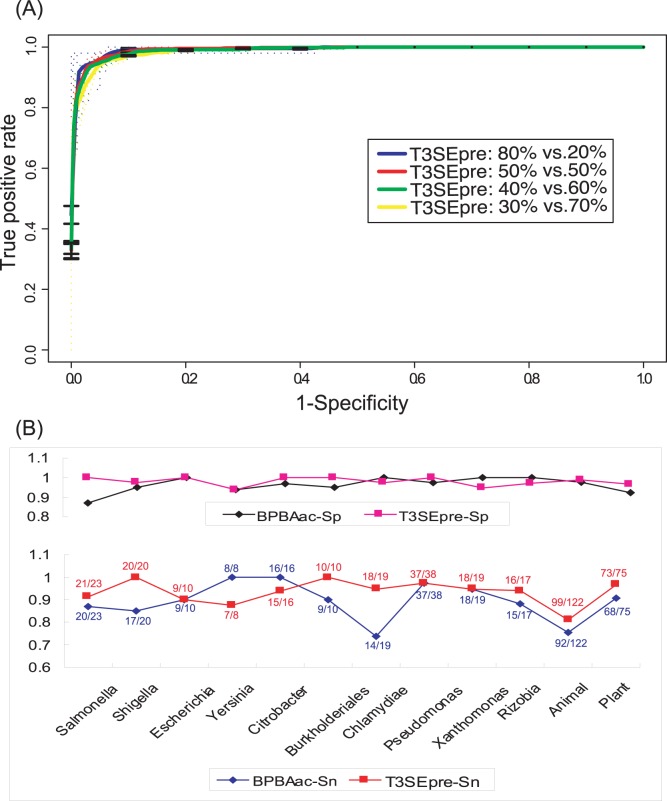
Stableness and inter-species applicability of T3SEpre. (A) ROC curves for T3SEpre models with different training to test data ratios. ‘Xx% vs. Yy%’ : ‘the percentage of training data versus that of testing data’. (B) Inter-species/group robustness of T3SEpre. Leave-One-Out strategy was adopted with the exception that, ‘One’ : data from ‘one species/group’. ‘Animal’ and ‘Plant’: ‘animal pathogens/symbionts’ and ‘plant pathogens/symbionts’, respectively. *Sn* and *Sp* represent sensitivity and specificity respectively. The recall rate of BPBAac and T3SEpre on each subgroup or species was indicated.

The robustness of T3SEpre was further examined using two strategies [21]: (1) Sub-datasets with different size were randomly selected from training data to re-train the model and to classify the remaining data; (2) Leave-One-Out strategy was adopted: the T3S and non-T3S proteins from one bacterial genus/subgroup was classified by the model trained on the remaining training data. The results showed that models trained by different sub-datasets performed equally well, and the performance was still fairly good even when only 30% of the original training data were used (Fig. 4A). In Leave-One-Out assessment, most of the effectors (93.4±5.4%) were recalled and consistently high specificity (98.0±2.2%) was obtained (Fig. 4B). A comparison was also made between T3SEpre and BPBAac. Except for few genera or subgroups (e.g., *Yersinia* and *Citrobacter*), T3SEpre recalled more (or identical number of) effectors at a similar high specificity (Fig. 4B). *Chlamydiae* is a genus phylogenetically distant to other bacteria with functional T3SS. Using effectors and non-effectors of other bacteria as training sequences, BPBAac recalled 73.7% (14/19) of *Chlamydiae* effectors; however, T3SEpre model trained with the same dataset recalled 94.7% (18/19) of the effectors (Fig. 4B). Results from animal and plant pathogens/symbionts’ T3S effectors also demonstrated the high efficacy of T3SEpre (Fig. 4B).

### 4. Stepwise Deletion Analysis for T3S Signal Sequences

**Figure 5:**
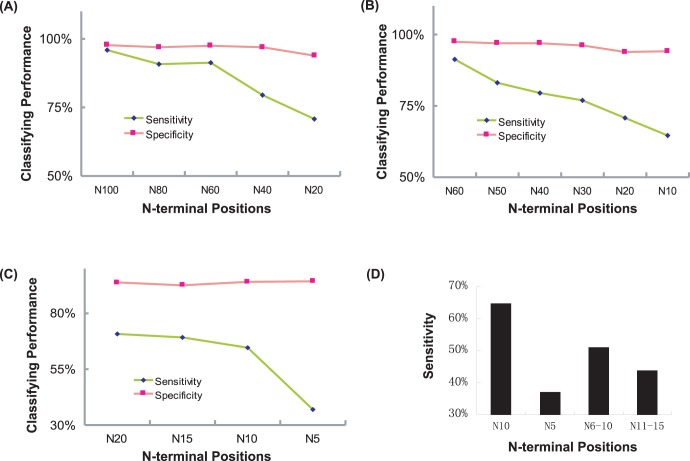
Performance of models with successively shortened N-terminal sequences. (A) The first 100, 80, 60, 40 and 20 amino acid positions. (B) The first 60, 50, 40, 30, 20 and 10 amino acid positions. (C) The first 20, 15, 10 and 5 amino acid positions. (D) 1–10, 1–5, 6–10 and 11–15 amino acid positions.

An in silico stepwise deletion analysis was designed to identify the most important positions contributing to the specificity of T3S signals. As shown in Fig. 5A, deletions of N-terminal positions 60–100 (N80 and N60) only slightly decreased the classifying performance of T3SEpre. When more positions were deleted, the recall rates were dramatically reduced for training models (N40 and N20). Starting from position 60 counting from the N-terminal end, the performance decreased with more deleted positions (N50-N10, Fig. 5B). Therefore, the N-terminal up to 60 amino acids form the most critical region for T3S signal.

Although with apparently decreased performance compared with N60, N20 can still recall 70% real T3S signals (Fig. 5C). Further deletions were then performed to delineate the important sub-regions within the N-terminal 20 positions. When C-terminal 5 and 10 positions were deleted from the N20 model (N15 and N10, respectively), the performance was not reduced apparently (Fig. 5C). However, the performance of the new model (N5) sharply decreased when more positions were deleted (Fig. 5C). Therefore, the N-terminal positions 6–10 may contain critical guiding features. The fact that model based on positions 6–10 (N6–10) performed apparently better than those based on adjacent positions with the same length (N5 and N11–15) further confirmed the importance of this short fragment (Fig. 5D). In fact, specific amino acid enrichments were apparently reflected in this short region (Fig. 1A, indicated in red rectangle). The Sse and Acc profiles in this region also showed striking enrichment of ‘helix’ and ‘exposed’, respectively (Fig. 1C and Fig. 1E, indicated in red rectangle). The joint features of Aac, Sse and Acc are also apparently different between T3S and non-T3S sequences (Fig. 2A–D).

### 5. Identification of New *Salmonella* T3S Effectors and Experimental Validation

A list of *Salmonella* T3S proteins (193 in total) were predicted using T3SEpre (Table S2), and most known effectors were correctly recalled (Table S2, in red and blue). Many newly predicted candidates include phage-originated proteins, or hypothetical proteins with unknown function (Table S2, in italic). Some proteins are known to be related with T3SS function, but it is not clear whether they can be translocated through T3SS conduit, e.g., invH and invE (Table S2, in green).

**Figure 6:**
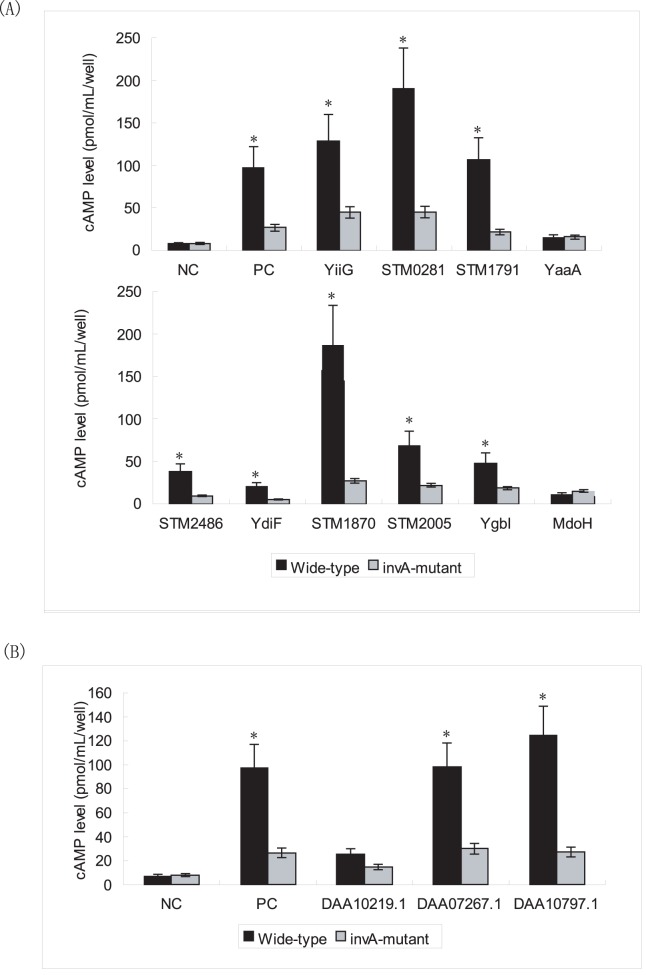
Translocation of predicted T3S proteins. (A) Cya translocation assays of *Salmonella* T3S protein candidates. Each construct was transformed into *Salmonella* SL1344 (Wild-type) and T3SS-deficient SL1344 strain (InvA-mutant). Duplicate was included for each test. Constructs pBADB-CyaA and pBADB-sipC-CyaA were used as negative control (NC) and positive control (PC), respectively. For each construct, *Student’s t* test was adopted to compare the cAMP level in the target wells co-incubated with the wild-type strain and InvA-mutant strain. Statistically significance was indicated by star (*p*<0.05). (B) Statistical analysis for Cya translocation assays of yeast T3S protein candidates. Constructs pBADB-CyaA and pBADB-sipC-CyaA were used as negative control (NC) and positive control (PC), respectively.

In total 36 candidate effectors were predicted with high score (≥0.5). Among them, 14 were known effectors and 22 were new predictions (Table 3). A large percentage (10/22, 45%) of the newly predicted T3S proteins were annotated with ‘unknown function’ (Table 3; shown in italic). We randomly selected 10 candidates for Cya translocation assay (Table 3; in bold). The assay result confirmed that 8 of them were translocated into co-cultured eukaryotic cells via *Salmonella* SPI-1 T3SS conduit (Fig. 6A). The other 2 candidates, mdoH and yaaA, were not secreted into the cytoplasm of eukaryotic cells (Fig. 6A). A microarray based gene co-expression analysis revealed that all the newly validated *Salmonella* T3S genes except STM2486 were co-expressed with invA under SPI-1 induction. They also showed high expression correlation with invA under 32 different culture conditions (Table S3).

**Table 3:** *Salmonella* T3S proteins predicted with T3SEpre (a strict cutoff, score > = 0.5, was used).

SeqID	Annotation	SVM_Value
Seq2779	sipC	2.06
Seq1359	sseG	1.83
Seq765	slrP	1.74
Seq1354	sseC	1.68
Seq1358	sseF	1.37
***Seq3891***	***yiiG***	***1.18***
Seq2778	sipD	1.13
Seq1989	sopA	1.02
Seq1355	sseD	1.00
Seq1794	sopE2	1.00
Seq1055	sopB	1.00
Seq1352	sseB	1.00
Seq1356	sseE	0.99
Seq1347	ssaB	0.98
*Seq4148*	*STM4312*	*0.95*
Seq1274	katE	0.92
Seq2774	sptP	0.90
Seq3446	ftsY	0.89
*Seq4253*	*STM4421*	*0.86*
Seq1681	tonB	0.85
***Seq272***	***STM0281***	***0.82***
Seq4195	hflK	0.75
Seq1973	pduO	0.72
**Seq1312**	**ydiF**	**0.71**
***Seq1809***	***STM1870***	***0.70***
***Seq1730***	***STM1791***	***0.70***
Seq2839	sopD	0.69
***Seq1931***	***STM2005***	***0.68***
*Seq2133*	*STM2209.1c*	*0.66*
**Seq2813**	**ygbI**	**0.65**
**Seq1111**	**mdoH**	**0.62**
Seq241	rcsF	0.62
Seq2764	orgC	0.61
***Seq5***	***yaaA***	***0.59***
***Seq2400***	***STM2486***	***0.57***
Seq400	nrdR	0.55

Among them, sipC, sipD, sopA, sopE2, sopB and sptP are known SPI-1 effectors while sseG, slrP, sseC, sseF, sseD, sseB and ssaB are known SPI-2 effectors.

### 6. Wide Distribution of T3S Signals in Different Species

Whole-genome T3S prediction was performed on a variety of micro-organsims, and a list of new T3S signal-containing candidates were identified. Interestingly, candidate T3S signal-containing genes were also predicted from species with no previously reported T3SSs, such as *Helicobacter* and *Mycobacterium* (Table 4 and Table S4). T3SSs have so far only been found in gram-negative bacteria, and yet a group of T3S candidates were confidently predicted with high scores from gram-positive bacteria and even in yeast (Table 4 and Table S4). To validate our prediction, 3 yeast candidates with high scores were selected for.Cya translocation assay. Interestingly, 2 of these 3 signal sequences could mediate translocation of Cya gene into eukaryotic cells via *Salmonella* SPI1 T3SS conduit (Fig. 6B).

**Table 4:** Potential T3S proteins in representative species predicted by T3SEpre (5 with highest prediction scores were given for each species).

Species	Protein	SVM_Value
**Agrobacterium** (NC_003062)	NP_353267.1	1.8262186013
(Gram −; no reported T3SS)	NP_353197.1	1.8182696817
	NP_354762.2	1.3438005288
	NP_353597.1	1.3053632004
	NP_529196.1	1.1505788212
**Helicobacter** (NC_000915)	NP_208221.1	1.2728691623
(Gram −; no T3SS)	NP_208270.1	0.81876863031
	NP_207400.1	0.79814466522
	NP_207364.1	0.76127749423
	NP_208245.1	0.74734863579
**Mycobacterium** (NC_002755)	NP_334701.1	2.5282200382
(Gram −; no T3SS)	NP_334584.1	2.1134425698
	NP_338544.1	2.091326393
	NP_337005.1	1.8061094469
	NP_337833.1	1.7706735128
**Staphylococcus** (NC_013450)	YP_003281316.1	1.1964289751
(Gram +; no T3SS)	YP_003282311.1	1.1653620221
	YP_003281905.1	0.99871685539
	YP_003281879.1	0.82115720581
	YP_003282635.1	0.70904028709
**Streptococcus** (NC_011900)	YP_002511184.1	0.86992793954
(Gram +; no T3SS)	YP_002510266.1	0.86910627471
	YP_002510762.1	0.73099842015
	YP_002511727.1	0.63822324187
	YP_002511008.1	0.60030423151
**Yeast** (S288c; no T3SS)	DAA10219.1	2.9246207038
	DAA07267.1	2.9232109626
	DAA10797.1	2.7428577459
	DAA09242.1	2.6276802073
	DAA08250.1	2.3665151381

## Discussion

### 1. Structural Features for T3S Protein Recognition

Several lines of evidence suggest that the N-terminal sequences contain signals guiding the specific recognition and secretion of T3S proteins [19]–[20], [31]–[34]. The molecular basis of this specificity, however, remains to be determined. Several groups attempted to find sequence-based specific T3S signal features. However, due to the high diversity of sequences, it is difficult to identify common domains or motifs within a certain bacterial genus or closely related genera [15], [35]. Recently, both sequence-based and position-based amino acid enrichment and depletion were discovered in the N-terminal region of T3S proteins [17], [21]. Computational models based on these features can well classify the T3S and non-T3S proteins, suggesting that the amino acid sequences at least encode part of the T3S specificity. Furthermore, some second-order elements including Sse and Acc were analyzed for more direct and specific features [17], [21]. Although differences were found between T3S and non-T3S proteins, these features were not considered as important for the specificity because they failed to improve the performance of classifier when incorporated independently [17], [21]. In this research, we treat the Aac, Sse and Acc features as inter-dependent co-variables and analyze the position-specific joint profiles of these features. We found that integration of these features apparently improved the classification power. Performance comparison between T3SEpre and BPBAac [21] showed that Sse and Acc are important features that contribute to the T3S-specificity. In a previous report, combining Aac, Sse and Acc did not particularly improve the model’s performance because they were treated as independent features [21]. We therefore believe that Sse and Acc features contribute to the specificity of T3S signals, and in an Aac-dependent manner.

We developed a ‘stepwise in silico deletion method’ to screen for the most important regions guiding specific type III secretion, and the N-terminal 6–10 positions were identified as the most critical motifs for such function (Fig. 5). The distinct amino acid composition, secondary structure, and solvent accessibility in this short region further indicate its significance. It is possible that non-continuous positions may jointly make important contribution.

The tertiary structure may directly explain the secretion specificity of T3S effectors. However, till now, the 3D structures have been resolved for only a limited number of T3S effectors, of which the N-terminal regions were mostly neglected because they are frequently disordered and very flexible [27]. We therefore adopted an in silico analysis strategy to predict their 3D structure (Materials and Methods). Among the 189 non-redundant T3S signal sequences, 41 were predicted with high confidence (Table S5; Zip file S1). The structure coordinates of these sequences were aligned against each other, and one fourth of them (11) were found to form a cluster that exhibits similar structure conformation: a loose N-terminal coil with varied length continued with multiple (3∼5) anti-paralleled helices or strands (Table S5 and Fig. S1A-E). This cluster should not be formed randomly because non-T3S sequences seldom adopt similar structure (Zip file S1). More interestingly, three sequences (*Yersinia* YopP, *EHEC* EspB, *Chlamydia* Q3KMQ0) showed nearly identical 3D structure even though no sequence similarity was found among them (Fig. S1B-E). There are also other sequences exhibiting high structure similarity, e.g., *Pseudomonas* HopPtoA1Pma, *Xanthomonas* XopD and *Vibrio* VopF, *Rhizobium* NopL and *Shigella* IpgB1, *Vibrio* VopC and *Pseudomonas* HopAN1, and *Ralstonia* RSc3401 and RSc1349, etc (Table S5 and Fig. S2A-D). This observation implied that the signal sequences of T3S proteins could possibly adopt special structural conformation to support their specific secretion. However, the 3D structures were only derived from computational prediction, which may not enough to draw decisive conclusion. Further experimental resolution of the T3S signal sequences is urgently required to unravel the mechanism of the T3S signal recognition.

### 2. Newly Identified *Salmonella* T3S Effectors

Using our high-performance computational model, 8 candidate T3S genes were identified from *Salmonella* genome. These genes are all co-expressed with T3SS apparatus genes under SPI-inducing conditions (Table S3). Except for STM2486, all the other newly validated T3S genes show clear co-expression with T3SS apparatus genes under different conditions (Table S3). Therefore, these genes potentially encode new effectors translocated into the host cytoplasm. Of the newly identified T3S candidates, 5 (yiiG, STM0281, STM1870, STM1791, STM2005 and STM2486) are annotated as hypothetical proteins with unknown function. These genes provide useful targets for further functional studies. Apart from the validated genes, a list of other T3S candidates were also predicted from *Salmonella* (Table 3 and Table S2). These potential *Salmonella* T3S effectors remain to be validated experimentally.

### 3. The Formation and Evolution of T3S Signal Sequences

In addition to the specific features embedded in the T3S signal sequences, how these sequences are formed and evolved also remains an enigma. In many bacterial species, some T3S effectors were resulted from horizontal gene transfer event together with T3SS apparatus [1]. For these effectors, the signal sequences seem to co-evolve with T3SS apparatus genes. However, more effectors were found to be scattered in the bacterial genomes. In model species such as *Salmonella*, it is known that different effectors function coordinately in the host-bacteria interactions [36]. It is interesting to investigate how these scattered effectors can be co-coordinately regulated.

Inspired by the ‘terminal re-assortment’ hypothesis proposed by Stavrinides et al. [37], a full-length T3S protein was partitioned into 2 parts: the N-terminal signal part and the C-terminal function part. We found that among the T3S proteins predicted with high scores from *Salmonella*, some are not co-expressed with either SPI-1 or SPI-2 apparatus genes (Data not shown). Besides, T3S signal-containing genes are also predicted from gram-positive bacteria and even yeasts (Table 4 and Fig. 6B). We therefore hypothesize that the T3S signal may form randomly and evolve independently with T3SS apparatus. A protein with putative T3S signals is not necessarily an effector because T3S effector must contain a functional domain and must be co-regulated with T3SS apparatus as well as other relevant genes for expression. For this reason, the candidate genes with T3S signals which are not co-expressed with corresponding T3SS apparatus, or those predicted from gram-positive bacteria or yeasts should not be called T3S effectors.

### 4. The Application of T3SEpre Software

Similar to BPBAac, T3SEpre is also an SVM classifier for T3S effector prediction [3], [21]. Both tools adopt a Bi-Profile Bayes (BPB) model to extract maximum likelihood-based position-specific features [21], [28]. The major difference between T3SEpre and BPBAac lies in the features: T3SEpre takes into account the secondary structures, solvent accessibility and amino acid composition of T3S signal regions while BPBAac only considers the amino acid composition features [21]. Compared with other software, such as SIEVE [24], Effective T3 [17], SSE-ACC [25], T3_MM [38], etc., which mostly extracts the sequence-based features, the uniqueness of T3SEpre is using position-specific instead of sequence-based features. Because each of these software tools adopts different molecular properties of T3S effectors or signal regions, a combination of two or more software is suggested to help increase the prediction accuracy.

## Materials and Methods

### Data Source

The source, homology-filtering and other handling procedures for positive (T3S) and negative (non-T3S) training datasets were similar to those described previously [21]. T3S proteins were annotated from literature with experimental evidence, while non-T3S proteins were randomly selected from the remained genes from different bacteria. For T3S and non-T3S proteins, only one representative was selected as the training sequence for each homologous cluster. JAligner (http://jaligner.sourceforge.net/) was used to identify homologous clusters with a sensitive pairwise/self ratio cutoff of 0.15 [17], [21]. In total, 189 and 385, non-redundant, T3S and non-T3S proteins were included in the final training datasets, respectively (Text S1). The Sse (represented as a combination sequence of ‘C’, ‘H’ or ‘E’) was predicted using PSIPRED [39], while SCRATCH [40] was used to predict the Acc (a combination of ‘B’ or ‘E’). The resulting positive and negative training datasets were pooled as the final training datasets and were randomly split into 5 subsets, each with equal number of items (T3S and non-T3S proteins as well the total number) for 5-fold cross-validation.

### Three-dimensional Structure Modelling and Comparison

I-TASSER and MUFOLD, two different high-accurate tertiary structure computational tools were adopted to predict structure for the N-terminal up to 100 amino acids of T3S and non-T3S proteins [41]–[42]. For each peptide sequence, MultiProt, a multiple protein structure alignment tool, was used to evaluate the consistency of structures predicted by I-TASSER and MUFOLD [43]. The high confident three-dimensional structure was included for further analysis only when it meets both of the following two criteria: (1) TM-score larger than 0.4 for I-TASSER prediction [41]; (2) high conformation similarity by I-TASSER and MUFOLD prediction based on MultiProt alignment results. The high-confident structures were further compared or clustered according to pairwise or multiple structure alignment by MultiProt, respectively. A cluster was identified when the grouped peptides share a structure similarity not smaller than 50% coordinates, and then compared by hand. All the structure alignments were performed with a sequence-ordered mode and an accuracy of 10 angstroms [43]. RasWin was used to view the 3D structures [44]. The PDB files for T3S and non-T3S sequence predictions are available upon request.

### Joint Feature Extraction, Model Training and Performance Comparison

Let vector S = {s*_1_*,s*_2_*,s*_3_*,…,s*_n_*} denotes a sequence of peptides, in which s represents amino acid while *1*, *2* … or *i* represents position and *n* represents total length of the sequence. For any *1*≤ *i* ≤ *n*, s*_i_* has 20 alternatives since it could be any one of the 20 amino acids. Let Sse[s*_i_*] and Acc[s*_i_*] represent the secondary structure element (Sse) and solvent accessibility state (Acc) that s*_i_* takes, respectively. Sse[s*_i_*] belongs to set {C, H, E} and Acc[s*_i_*] belongs to set {B, E}, and consequently for any position *_i_* (*_1_*≤ *_i_* ≤ *_n_*), there are 20×3×2 = 120 types of combination of the three categories of components (amino acid, Sse and Acc). The frequency of each type of combination was calculated for each position of positive training sequences (T3S) and negative training sequences (non-T3S), represented as P_+1_(s*_i_*Sse[s*_i_*]Acc[s*_i_*]) and P_-1_(s*_i_*Sse[s*_i_*]Acc[s*_i_*]), respectively. For each sequence, a feature vector containing 2*n* bi-profile frequencies was obtained for n sequential positions (*n* was set as 100 in this research):
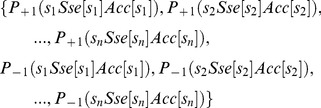
(1)


The bi-profile features from both positive and negative samples were extracted with a bi-profile model [21], [28], followed by training with a support vector machine (SVM). Radial basis kernel function 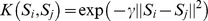 was selected for SVM prediction. SVM parameter γ and penalty parameter *C* were optimized using grid search based on 10-fold cross-validation [45].

SSE-ACC, BPBAac and BPBAll were re-trained with the same dataset prepared in this study with prior parameters suggested by the original paper and the 10-fold cross-validation grid searching results. The performance was compared among different software based on a 5-fold cross-validation evaluation.

The parameters for performance assessment, including Accuracy (*A*), Specificity (*Sp*), Sensitivity (*Sn*), Receiver Operating Characteristic (*ROC*) curve, the area under *ROC* curve (*AUC*) and Matthews Correlation Coefficient (*MCC*), were well defined in Wang et al, 2011 [21].

### Stepwise *in silico* Deletion Analysis

For each step, peptide strings with a defined length were deleted successively at a given direction (C- or N-terminal end) for all the training sequences. The successively shortened sequences were used as a new training dataset to train the model. The performance of each new model was evaluated by *Sn*, *Sp* and *AUC*, all of which were assessed by average results for 5-fold cross validations. The deleted length was set as a series of 20 amino acids at the beginning, followed by 10 amino acids, and 5 amino acids respectively.

### Performance Comparison among Different Software

Apart from the training dataset used in this study, four other independent datasets containing validated T3S effectors and control proteins were included: Arnold 2009 [17], Lower 2009 [18], Mukaihara 2010 [29], and Baltrus 2011 [30] (Text S2, S3, S4, and 5). The Mukaihara 2010 data were also used in Wang *et al*., 2011, in which only one protein was randomly selected from a homologous cluster while the rest were removed. Therefore, the final number of included effectors was 35 although the actual number identified in the original study was 46. The N-terminal 100 amino acid (not including the starting ‘M’) sequences were used to predict the secondary structure and solvent accessibility as described before.

The original parameters were adopted for BPBAac and Effective T3 to classify the proteins of test datasets (default decision value 0.5 and 0.99 for BPBAac and Effective T3 respectively) [17], [21].

### Implementation of T3SEpre and Whole-genome T3S Protein Prediction

The T3SEpre software was written in Perl and R. An R package for T3SEpre can be freely downloaded from http://biocomputer.bio.cuhk.edu.hk/softwares/T3SEpre. A web server was also developed to implement on-line prediction of T3SEpre. The interface was developed with HTML, PHP and Javascript (http://biocomputer.bio.cuhk.edu.hk/T3DB/T3SEpre.php). Currently, both the stand-alone software and the web server require users to predict Sse and Acc features with corresponding software before implementation of T3SEpre. Details (including parameter selection) about the usage can be found within the package or web server documents.

Bacteria or yeast whole-genome protein sequences were downloaded from NCBI Genome database. The N-teminal up to 100 amino acid position or full-length sequence for peptides with fewer than 100 amino acids was extracted for secondary structure prediction using PSIPRED [39]. The solvent accessibility was predicted using SCRATCH [40]. The amino acid sequence, Sse sequence, and Acc sequence were used together for T3SEpre to predict if the corresponding peptide contains T3S signals. For more specific results, a default cutoff value of 0.5 was used.

### Bacteria, Plasmids and Cell Lines

*E.coli* DH5alpha and *Salmonella typhimurium* strain SL1344 were used in this research. SL1344 was obtained from Salmonella Genetic Stock Centre (SGSC, http://www.ucalgary.ca/~kesander). SPI1 T3SS deficient SL1344 strain was constructed by disrupting invA gene using a gene replacement method [46]. The bacteria were cultured on LB plate or in LB broth with or without 100 mg/L ampicilin. The yeast genome DNA was provided by Mr Gao Caiji from the Chinese University of Hong Kong. The plasmids used in this study were summarized in Fig. S3 and Table S6. The pMS107 plasmid with Bordetella CyaA gene insertion was gifted by Professor Guy R Cornelis (Focal Area Infection Biology, Biozentrum, University of Basel, Switzerland). A pair of primers (Table S7) were designed to PCR amplify CyaA gene. The pBADB-Myc-His plasmid with an L-arabinose-induced promoter and C-terminal Myc and His double tags, was ordered from Invitrogen (Cat. No. V440-01). CyaA gene fragment was cloned into pBADB-Myc-His plasmid, generating pBADB-CyaA-tag (Fig. S3). DNA sequences encoding N-terminal 100 amino acids of candidate T3S proteins were amplified and cloned into pBADB-CyaA-tag at the 5′ end of CyaA sequence, resulting in different constructs (Table S6 and Table S7).

Human liver cancer HepG2 cells were cultured in DMEM supplemented with 10% fetal bovine serum. Cells were grown at 37°C in a 5% CO2 humidified incubator.

### Western Blotting and Cya Translocation Assay

Wild-type and invA-mutant SL1344 strains transfected with different constructs were cultured for 12 h in LB-0.3 M NaCl medium containing 100 mg/L ampicilin. The culture was diluted 1∶100 fold using fresh LB-0.3 M NaCl medium, and grown for another 3 h under slow agitation to obtain an optical density of OD600 0.8∼0.9 (Salmonella Pathogenecity Island 1 (SPI-1) inducing conditions). The fusion proteins with pBAD promoter were induced with 20% L-arabinose during the last 3 hours. Bacterial total proteins were extracted and re-suspended in sodium dodecyl sulfate-polyacrylamide gel electrophoresis (SDS-PAGE) sample buffer for SDS-PAGE analysis. Protein expression was detected using Western blotting with anti-myc antibody (Invitrogen, Cat. No. R950-25).

The mechanism of Cya translocation assay was shown in Fig. S4 [47]. HepG2 cells were plated into 24-well tissue culture plates 1 day before infection. Each well contains 1 ml medium, and after 24 h culture, the density of adherent cells reached 2×10ˆ5 cells per well. HepG2 cells were washed twice, replaced with fresh medium, and used to infect *Salmonella* for 2 hour at a multiplicity of infection (MOI) of 20 [48]. After infection, the cells were washed with ice-cold Phosphate-buffered saline (PBS) for three times, and then lysed in 100 ul of extraction solution (50 mN HCl/0.1% Triton×100) on ice. The lysate was boiled in a water bath for 5 min, followed by neutralization with 6 ul of 0.5 M NaOH. cAMP was extracted with ethanol. After centrifugation at 11500×g for 5 min, the supernatant containing cAMP was lyophilized and then quantified using a cAMP ELISA kit (R&D, Cat. No. KGE002B).

### Microarray Dataset and Data Analysis

Microarray dataset GSE2456 profiling the expression of *Salmonella* genes under 32 different growth conditions was downloaded from NCBI GEO database. All the gene chips used the same platform and the experiments were performed at the same time by the same group (McClelland laboratory, unpublished). After normalization, the expression values for each gene were analyzed for their expression correlations and co-expression with both SPI-1 (invA, invG and invC, encoding export apparatus, outer membrane ring and ATPase, respectively) and SPI-2 (ssaV, ssaC and ssaN, encoding export apparatus, outer membrane ring and ATPase, respectively) apparatus genes. For expression level correlation analysis, Pearson Correlation Coefficients (PCCs) and Spearman Rank Correlation Coefficients (SCCs) were calculated. To analyze co-expression between candidate and SPI-1 or SPI-2 apparatus genes, expression levels for each gene were observed and ranked. Four categories were defined: “+++” represents the situation that expression of target gene is strongly co-induced under InvA strongly-inducing conditions (the expression level of target gene ranking top 10%); “++” represents the situation that expression of target gene is relatively strongly induced under InvA strongly-inducing conditions (ranking top 20%); “+” represents the situation that expression of target gene is induced under InvA strongly-inducing conditions (ranking top 50%); otherwise, the target gene is considered not co-expressed under SPI-1 inducing conditions, and is represented as ‘-’.

## Supporting Information

Figure S1 **A common 3D structure cluster of T3S signal sequences and similar structures.** (A) The cluster (11 sequences) contains common 3D structure. (B) Structure alignment among *Yersinia* YopP, *EHEC* EspB, *Chlamydia* Q3KMQ0, and *Shigella* VirA signal sequences; (C)–(E) Structure and topology of *Yersinia* YopP, *EHEC* EspB, *Chlamydia* Q3KMQ0, respectively. The backbones of aligned peptides were shown in (A) and (B), while strands for individual peptide were shown in (C)–(E). N-termini were shown in blue and C-termini in red.
(PDF)

Figure S2 **Structure alignments for T3S signal sequences with similar 3D structures.** *Pseudomonas* HopPtoA1Pma, *Xanthomonas* XopD and *Vibrio* VopF; (B) *Rhizobium* NopL and *Shigella* IpgB1; (C) *Vibrio* VopC and *Pseudomonas* HopAN; (D) *Ralstonia* RSc3401 and RSc1349. Structure backbones were shown for the aligned peptides. N-termini were shown in blue and C-termini were in red.
(PDF)

Figure S3 **Construction of Cya translocation reporter plasmid.** Plasmid pMS107 containing CyaA fragment was used as template to amplify CyaA gene with EcoRI and XhoI restriction sites. The PCR product was further cloned into plasmid pBADB-Myc-His to get the resulting pBADB-CyaA-tag reporter plasmid. Candidate signal sequences were cloned into pBADB-CyaA-tag plasmid between XbaI and EcoRI sites to obtained different testing plasmids, respectively.
(PDF)

Figure S4 **Principles of CyaA translocation assay.** CyaA reporter plasmids inserted with N-terminal candidate signal sequences were transformed into bacteria of functional T3SSs. Under induction of L-arabinose, the mosaic protein fused with N-terminal candidate T3S signals, CyaA polypeptides, and C-terminal Myc-His double tags will be expressed. Under T3SS induction conditions, T3SS apparatus genes will be expressed and assembled. If the signal sequence cloned in reporter plasmid is true T3S signal, it will be specifically recognized by T3SS apparatus, and consequently the fusion protein will be translocated into contacting eukaryotic cells. In cytoplasm of eukaryotic cells, with the assistance of Calmodulin (CaM) protein, CyaA protein will exert its function to catalyze the reaction by which ATP is changed to cAMP. Therefore, the cAMP level will be increased significantly.
(PDF)

Table S1 Joint feature profiles in the N-terminal up to 50 positions.
(XLS)

Table S2 Salmonella T3S effectors predicted by T3SEpre.
(XLS)

Table S3 Expression correlation with InvA and co-expression under InvA inducing conditions of newly identified Salmonella T3S proteins.
(DOC)

Table S4 T3S signal-containing proteins predicted from organisms without reported T3SS.
(XLS)

Table S5 3D structure predictions and comparison for T3S signal sequences.
(DOC)

Table S6 Plasmids used in this study.
(DOC)

Table S7 Primers used in this study.
(DOC)

Text S1 **Training peptide, secondary structure and solvent accessibility.**
(TXT)

Text S2 **Arnold 2009 training sequences.**
(TXT)

Text S3 **Lower 2009 training_sequences.**
(TXT)

Text S4 **Mukaihara 2010 Ralstonia T3S proteins.**
(TXT)

Text S5 **Baltrus 2011 Comprehensive Pseudomonas T3S protein.**
(TXT)

Zip file S1 **Predicted 3D structure of T3S and non-T3S signal sequences.**
(ZIP)

## References

[1] Hueck CJ (1998) Type III protein secretion systems in bacterial pathogens of animals and plants. Mol. Biol. Rev. 62: 379–433. 10.1128/mmbr.62.2.379-433.1998 PMC98920 9618447

[2] Ghosp P (2004) Process of protein transport by the type III secretion system. Microbiol. Mol. Biol. Rev. 68: 771–795. 10.1128/MMBR.68.4.771-795.2004 PMC539011 15590783

[3] Wang Y, Huang H, Sun M, Zhang Q, Guo D (2012) T3DB: an integrated database for bacterial Type III Secretion System. BMC Bioinformatics 13: 66. 22545727 10.1186/1471-2105-13-66 PMC3424820

[4] Galán JE (2009) Common themes in the design and function of bacterial effectors. Cell. Host Microbe. 5: 571–9. 10.1016/j.chom.2009.04.008 PMC2729653 19527884

[5] Lindeberg M, Collmer A (2009) Gene ontology for type III effectors: capturing processes at the host-pathogen interface. Trend. Microbiol. 17: 304–311. 10.1016/j.tim.2009.04.001 19576777

[6] Galán JE, Curtiss R 3rd (1989) Cloning and molecular characterization of genes whose products allow Salmonella typhimurium to penetrate tissue culture cells. Proc. Natl. Acad. Sci. USA 86: 6383–7. 10.1073/pnas.86.16.6383 PMC297844 2548211

[7] Jarvis KG, Girón JA, Jerse AE, McDaniel TK, Donnenberg MS, et al (1995) Enteropathgenic Escherichia coli contains a putative type III secretion system necessary for the export of proteins involved in attaching and effacing lesion formation. Proc. Natl. Acad. Sci. USA 92: 7996–8000. 10.1073/pnas.92.17.7996 PMC41273 7644527

[8] Huang HC, Lin RH, Chang CJ, Collmer A, Deng WL (1995) The complete hrp gene cluster of Pseudomonas syringae pv. syringae 61 includes two blocks of genes required for harpinPss secretion taht are arranged colinearly with Yersinia ysc homologs. MPMI, 8, 733–746. 10.1094/mpmi-8-0733 7579617

[9] Hong KH, Miller VL (1998) Identification of novel Salmonella invasion locus homologous to Shigella ipgDE. J. Bacteriol (180) 1793–1802. 10.1128/jb.180.7.1793-1802.1998 PMC107092 9537377

[10] Noël L, Thieme F, Nennstiel D, Bonas U (2002) Two novel type III-secreted proteins of Xanthomonas campestris pv. vescatoria are encoded within the hrp pathogenicity island. J. Bacteriol. 184: 1340–1348. 10.1128/JB.184.5.1340-1348.2002 PMC134860 11844763

[11] Noël L, Thieme F, Gäbler J, Büttner D, Bonas U (2003) XopC and XopJ, two novel type III effector proteins from Xanthomonas campestris pv. vescatoria. J. Bacteriol. 185: 7092–7102. 10.1128/JB.185.24.7092-7102.2003 PMC296255 14645268

[12] Kaniga K, Trollinger D, Galán JE (1995) Identification of two targets of the type III protein secretion system system encoded by the inv and spa loci of Salmonella typhymurium that have homology to the Shigella IpaD and IpaA proteins. J.Bacteriol. 177: 7078–7085. 10.1128/jb.177.24.7078-7085.1995 PMC177584 8522512

[13] Hardt WD, Galán JE (1997) A secreted Salmonella protein with homology to an avirulence determinant of plant pathogenic bacteria. Proc. Natl. Acad. Sci. USA. 94: 9887–9892. 10.1073/pnas.94.18.9887 PMC23287 9275221

[14] Panina EM, Mattoo S, Griffith N, Kozak NA, Yuk MH, et al (2005) A genome-wide screen identifies a Bordetella type III secretion effector and candidate effectors in other species. Mol. Microbiol. 58: 267–279. 10.1111/j.1365-2958.2005.04823.x 16164564

[15] Petnicki-Ocwieja T, Schneider DJ, Tam VC, Chancey ST, Shan L, et al (2002) Genomewide identification of proteins secreted by the Hrp type III protein secretion system of Pseudomonas syringae pv. DC3000. Proc. Natl. Acad. Sci. USA 99: 7652–7657. 10.1073/pnas.112183899 PMC124312 12032338

[16] Tobe T, Beatson SA, Taniguchi H, Abe H, Bailey CM, et al (2006) An extensive repertoire of type III secretion effectors in Escherichia coli O157 and the role of lambdoid phages in their dissemination. Proc. Natl. Acad. Sci. USA 103: 14941–14946. 10.1073/pnas.0604891103 PMC1595455 16990433

[17] Arnold R, Brandmaier S, Kleine F, Tischler P, Heinz E, et al (2009) Sequence-based prediction of type III secreted proteins. PLoS pathogens 5: e1000376. 19390696 10.1371/journal.ppat.1000376 PMC2669295

[18] Löwer M, Schneider G (2009) Prediction of type III secretion signals in genomes of gram-negative bacteria. PLoS ONE 4: e5917. 19526054 10.1371/journal.pone.0005917 PMC2690842

[19] Rüssmann H, Kubori T, Sauer J, Galán JE (2002) Molecular and functional analysis of the type III secretion signal of the Salmonella enterica InvJ protein. Mol. Microbiol. 46: 769–779. 10.1046/j.1365-2958.2002.03196.x 12410834

[20] Lloyd SA, Norman M, Rosqvist R, Wolf-Watz H (2001) Yersinia YopE is targeted for type III secretion by N-terminal, not mRNA, signals. Mol. Microbiol. 39: 520–531. 10.1046/j.1365-2958.2001.02271.x 11136471

[21] Wang Y, Zhang Q, Sun MA, Guo D (2011) High-accuracy prediction of bacterial type III secreted effectors based on position-specific amino acid composition profiles. Bioinformatics 27: 777–784. 21233168 10.1093/bioinformatics/btr021

[22] Rüssmann H, Igwe EI, Sauer J, Hardt WD, Bubert A, et al (2001) Protection against murine listeriosis by oral vaccination with recombinant Salmonella expressing hybrid Yersinia type III proteins. J. Immunol. 167: 357–365. 10.4049/jimmunol.167.1.357 11418671

[23] Girard F, Crepin VF, Frankel G (2009) Modelling of infection by enteropathogenic Escherichia coli strains in lineages 2 and 4 ex vivo and in vivo by using Citrobacter rodentium expressing TccP. Infect. Immun. 77: 1304–1314. 10.1128/IAI.01351-08 PMC2663131 19188355

[24] Samudrala R, Heffron F, McDermott JE (2009) Accurate prediction of secreted substrates and identification of a conserved putative secretion signal for type III secretion systems. PLoS pathogens 5: e1000375. 19390620 10.1371/journal.ppat.1000375 PMC2668754

[25] Yang Y, Zhao J, Morgan RL, Ma W, Jiang T (2010) Computational prediction of type III secreted proteins from gram-negative bacteria. BMC bioinformatics (Suppl 1): S47. 10.1186/1471-2105-11-S1-S47 PMC3009519 20122221

[26] Arnold R, Jehl A, Rattei T (2010) Targeting effectors: the molecular recognition of Type III secreted proteins. Microb. Infect. 12: 346–358. 10.1016/j.micinf.2010.02.003 20178857

[27] Galán JE, Wolf-Watz H (2006) Protein delivery into eukaryotic cells by type III secretion machines. Nature 444: 567–73. 17136086 10.1038/nature05272

[28] Shao J, Xu D, Tsai SN, Wang Y, Ngai SM (2009) Computational identification of protein methylation sites through bi-profile Bayes feature extraction. PloS one 4: e4920. 19290060 10.1371/journal.pone.0004920 PMC2654709

[29] Mukaihara T, Tamura N, Iwabuchi M (2010) Genome-wide identification of a large repertoire of Ralstonia solanacearum type III effector proteins by a new functional screen. MPMI 23: 251–262. 20121447 10.1094/MPMI-23-3-0251

[30] Baltrus DA, Nishimura MT, Romanchuk A, Chang JH, Mukhtar MS, et al (2011) Dynamic Evolution of Pathogenicity Revealed by Sequencing and Comparative Genomics of 19 Pseudomonas syringae Isolates. *PLoS Pathog* 7(7): e1002132. 21799664 10.1371/journal.ppat.1002132 PMC3136466

[31] Karavolos MH, Roe AJ, Wilson M, Henderson J, Lee JJ, et al (2005) Type III secretion of the Salmonella effector protein SopE is mediated via an N-terminal amino acid signal and not an mRNA sequence. J. Bacteriol. 187: 1559–1567. 10.1128/JB.187.5.1559-1567.2005 PMC1064012 15716426

[32] Lloyd SA, Sjöström M, Andersson S, Wolf-Watz H (2002) Molecular characterization of type III secretion signals via analysis of synthetic N-terminal amino acid sequences. Mol. Microbiol. 43: 51–59. 10.1046/j.1365-2958.2002.02738.x 11849536

[33] Schechter LM, Roberts KA, Jamir Y, Alfano JR, Collmer A (2004) Pseudomonas syringae type III secretion system targeting signals and novel effectors studied with a Cya translocation reporter. J Bacteriol. 186: 543–555. 10.1128/JB.186.2.543-555.2004 PMC305754 14702323

[34] Wang Y, Hou Y, Huang H, Liu GR, White AP, et al (2008) Two oral HBx vaccines delivered by live attenuated Salmonella: both eliciting effective anti-tumor immunity. Cancer Lett 263: 67–76. 18226855 10.1016/j.canlet.2007.12.022

[35] Buchko GW, Niemann G, Baker ES, Belov ME, Smith RD, et al (2010) A multi-pronged search for a common structural motif in the secretion signal of Salmonella enterica serovar Typhimurium type III effector proteins. Mol. Biosyst. 6: 2448–2458. 10.1039/c0mb00097c PMC3282560 20877914

[36] Kubori T, Galán JE (2003) Temporal regulation of salmonella virulence effector function by proteasome-dependent protein degration. Cell 115: 333–342. 14636560 10.1016/s0092-8674(03)00849-3

[37] Stavrinides J, Ma W, Guttman DS (2006) Terminal reassortment drives the quantum evolution of type III effectors in bacterial pathogens. PLoS. Pathog. 2: e104. 10.1371/journal.ppat.0020104 PMC1599762 17040127

[38] Wang Y, Sun M, Bao H, White AP (2013) T3_MM: a Markov Model effectively classifies bacterial Type III Secretion Signals. *PLoS ONE*. In press. 10.1371/journal.pone.0058173 PMC3589343 23472154

[39] McGuffin LJ, Bryson K, Jones DT (2000) The PSIPRED protein structure prediction server. Bioinformatics 16: 404–405. 10869041 10.1093/bioinformatics/16.4.404

[40] Cheng J, Randall AZ, Sweredoski MJ, Baldi P (2005) SCRATCH: a protein structure and structural feature prediction server. Nuceic Acids Res. 33: W72–W76. 10.1093/nar/gki396 PMC1160157 15980571

[41] Wu S, Skolnick J, Zhang Y (2007) Ab initio modelling of small proteins by iterative TASSER simulations. BMC Biol. 5: 17. 10.1186/1741-7007-5-17 PMC1878469 17488521

[42] Zhang J, Wang Q, Barz B, He Z, Kosztin I, et al.. (2010) MUFOLD: A new solution for protein 3D structure prediction. Proteins, 78, 1137–1152. 10.1002/prot.22634 PMC2885889 19927325

[43] Shatsky M, Nussinov R, Wolfson HJ (2004) A method for simultaneous alignment of multiple protein structures. Proteins 56: 143–156. 15162494 10.1002/prot.10628

[44] Sayle R, Milner-White EJ (1995) RASMOL: biomolecular graphics for all. Trends. Biochem. Sci. 20: 374. 10.1016/s0968-0004(00)89080-5 7482707

[45] Scholkopf B, Smola AJ (2002) Learning with Kernels. Cambridge: MIT Press.

[46] White AP, Collinson SK, Burian J, Clouthier SC, Banser PA, et al (1999) High efficiency gene replacement in *Salmonella enteritidis*: Chimeric fimbrins containing a T-cell epitope from Leishmania major. *Vaccine* 17(17): 2150–2161. 10367948 10.1016/s0264-410x(98)00491-5

[47] Sory MP, Cornelis GR (1994) Translocation of a hybrid YopE-adenylate cyclase from Yersinia enterocolitica into Hela cells. Mol. Microbiol. 14: 583–394. 10.1111/j.1365-2958.1994.tb02191.x 7885236

[48] Higashide W, Zhou D (2006) The first 45 amino acids of SopA are necessary for InvB binding and SPI-1 secretion. J. Bacteriol. 188: 2411–2420. 10.1128/JB.188.7.2411-2420.2006 PMC1428425 16547027

