# Supplementary figures and images for: Effective Identification of Bacterial Type III Secretion Signals Using Joint Element Features
Source: PLoS One. 2013 Apr 4;8(4):e59754. doi: 10.1371/journal.pone.0059754 (PMC3617162; doi:10.1371/journal.pone.0059754)

(A)

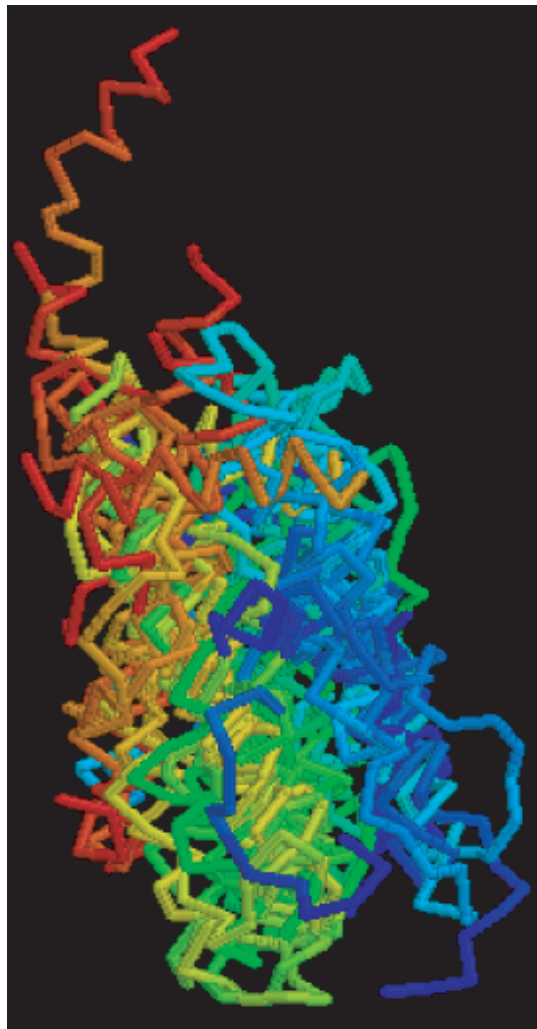

(B)

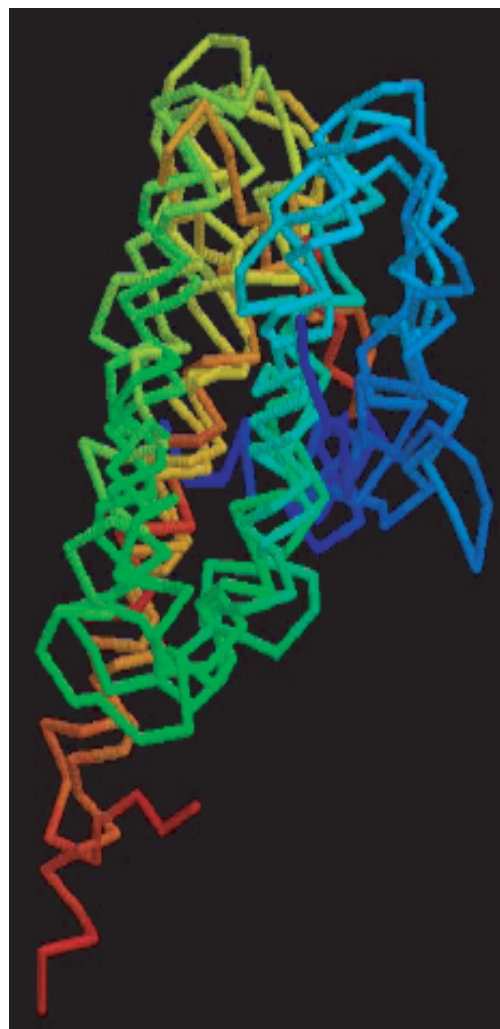

(C)

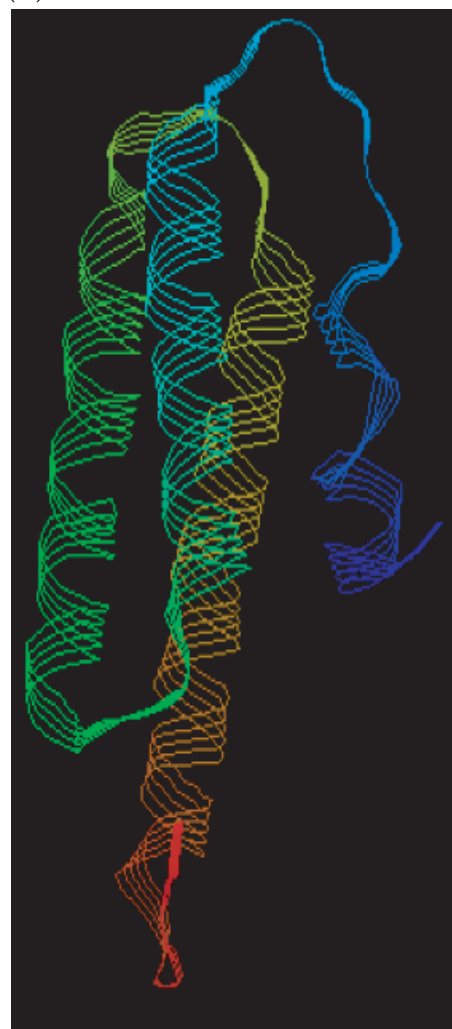

(D)

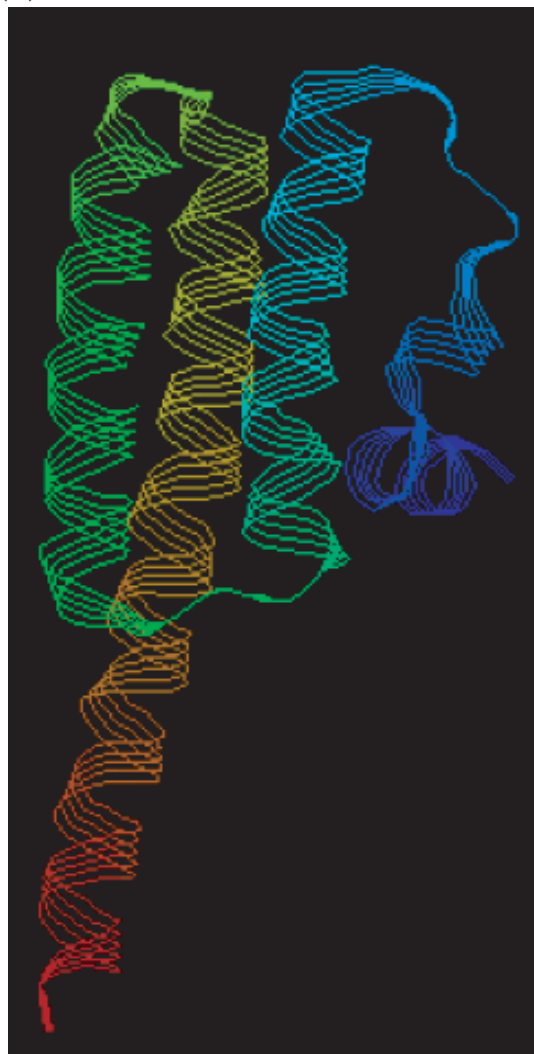

(E)

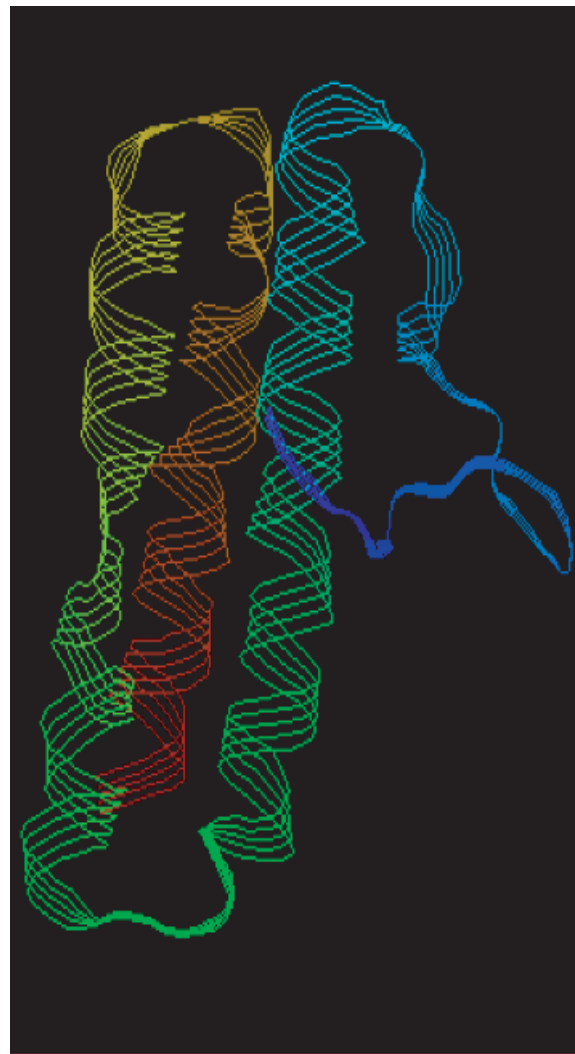

Supplement: Figure S1 — A common 3D structure cluster of T3S signal sequences and similar structures. (A) The cluster (11 sequences) contains common 3D structure. (B) Structure alignment among Yersinia YopP, EHEC EspB, Chlamydia Q3KMQ0, and Shigella VirA signal sequences; (C)–(E) Structure and topology of Yersinia YopP, EHEC EspB, Chlamydia Q3KMQ0, respectively. The backbones of aligned peptides were shown in (A) and (B), while strands for individual peptide were shown in (C)–(E). N-termini were shown in blue and C-termini in red. (PDF) [file pone.0059754.s001.pdf]

(A)

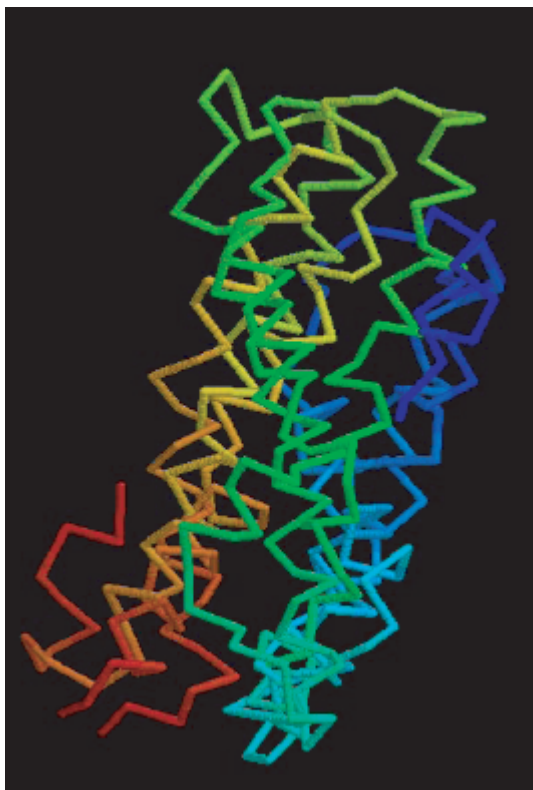

(B)

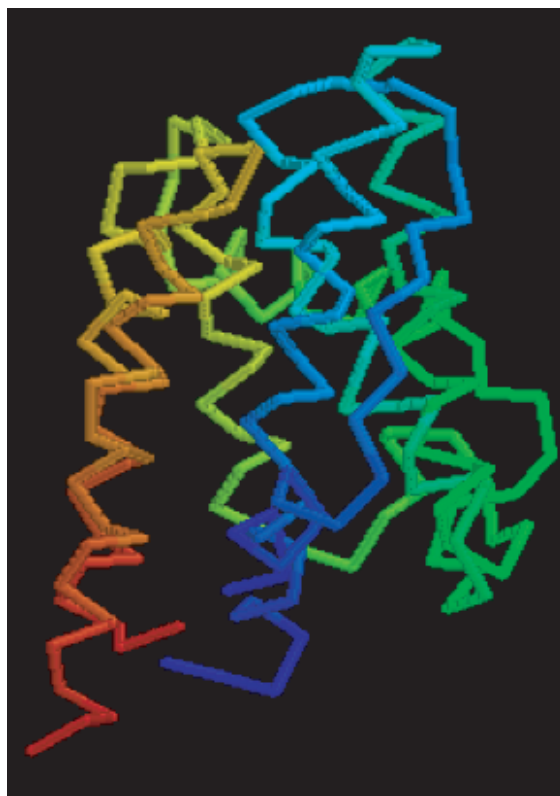

(C)

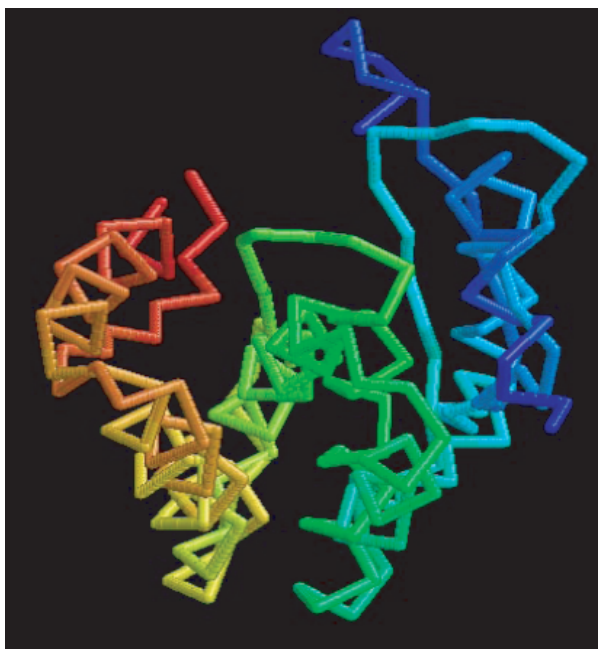

(D)

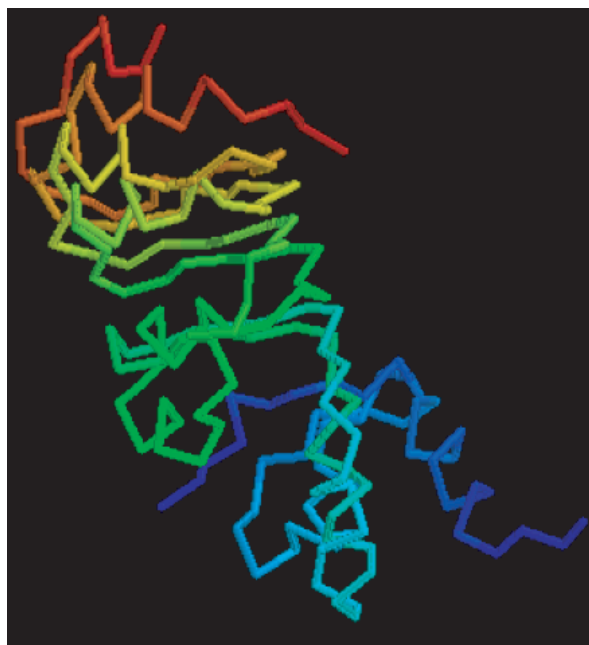

Supplement: Figure S2 — Structure alignments for T3S signal sequences with similar 3D structures. Pseudomonas HopPtoA1Pma, Xanthomonas XopD and Vibrio VopF; (B) Rhizobium NopL and Shigella IpgB1; (C) Vibrio VopC and Pseudomonas HopAN; (D) Ralstonia RSc3401 and RSc1349. Structure backbones were shown for the aligned peptides. N-termini were shown in blue and C-termini were in red. (PDF) [file pone.0059754.s002.pdf]

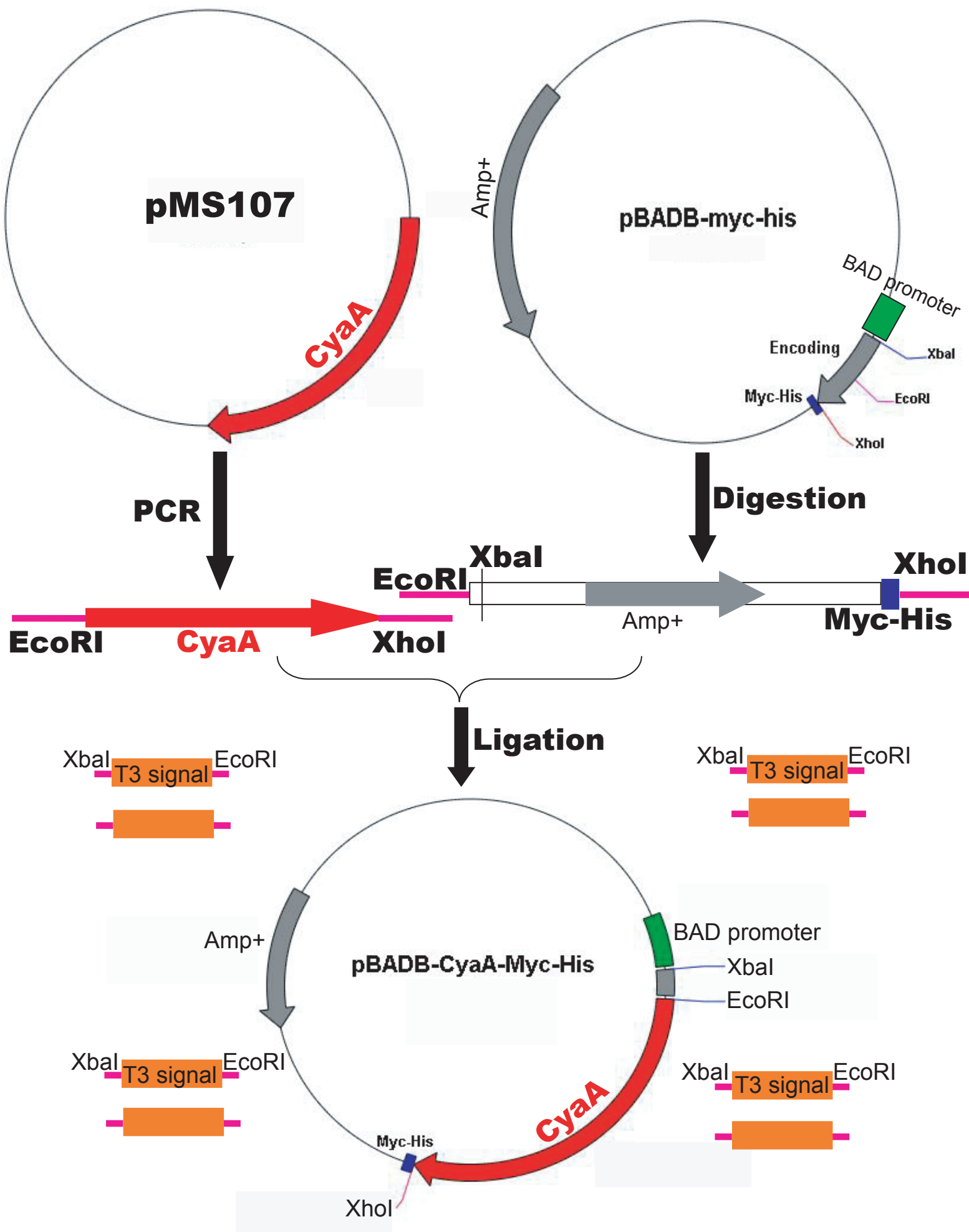

Supplement: Figure S3 — Construction of Cya translocation reporter plasmid. Plasmid pMS107 containing CyaA fragment was used as template to amplify CyaA gene with EcoRI and XhoI restriction sites. The PCR product was further cloned into plasmid pBADB-Myc-His to get the resulting pBADB-CyaA-tag reporter plasmid. Candidate signal sequences were cloned into pBADB-CyaA-tag plasmid between XbaI and EcoRI sites to obtained different testing plasmids, respectively. (PDF) [file pone.0059754.s003.pdf]

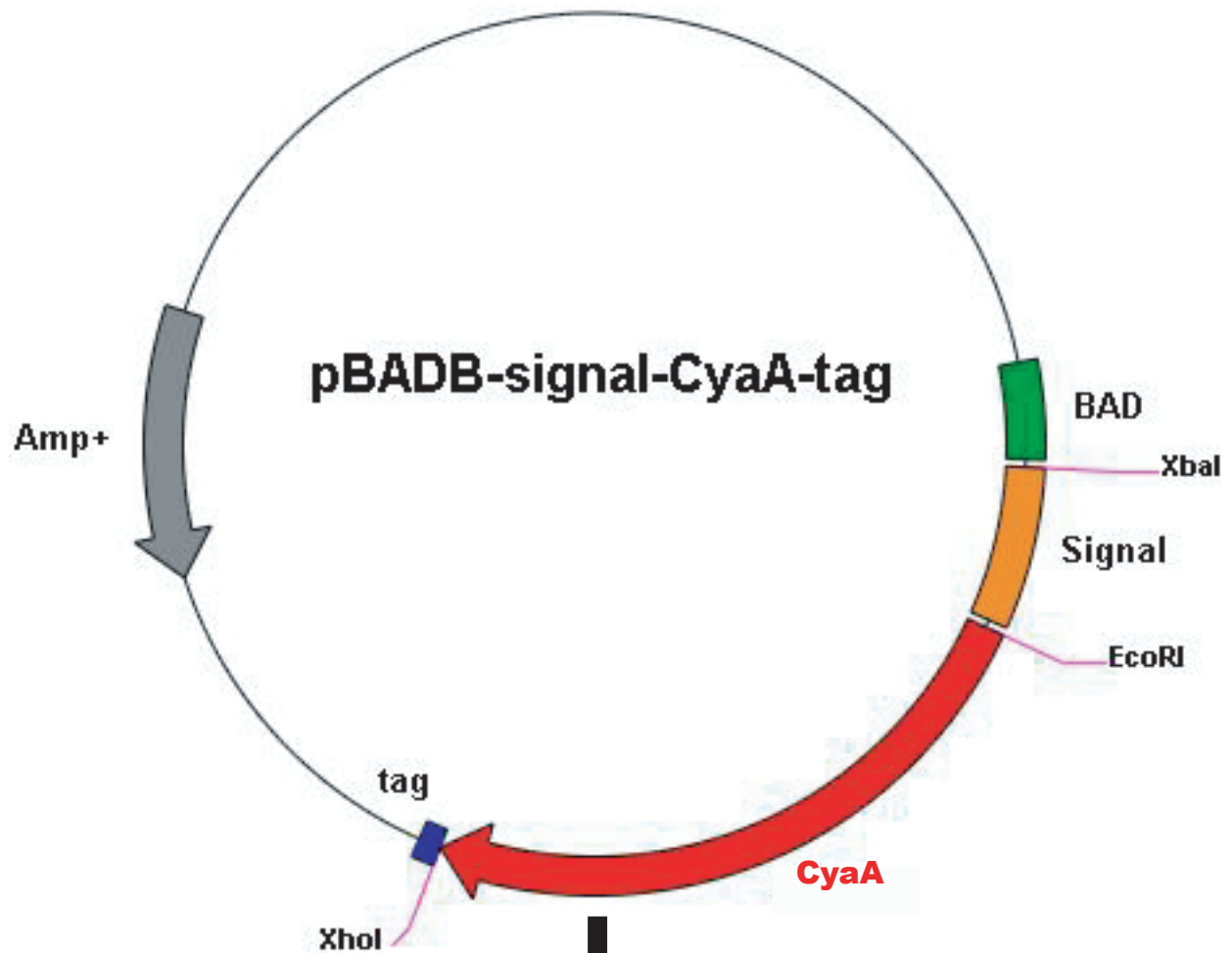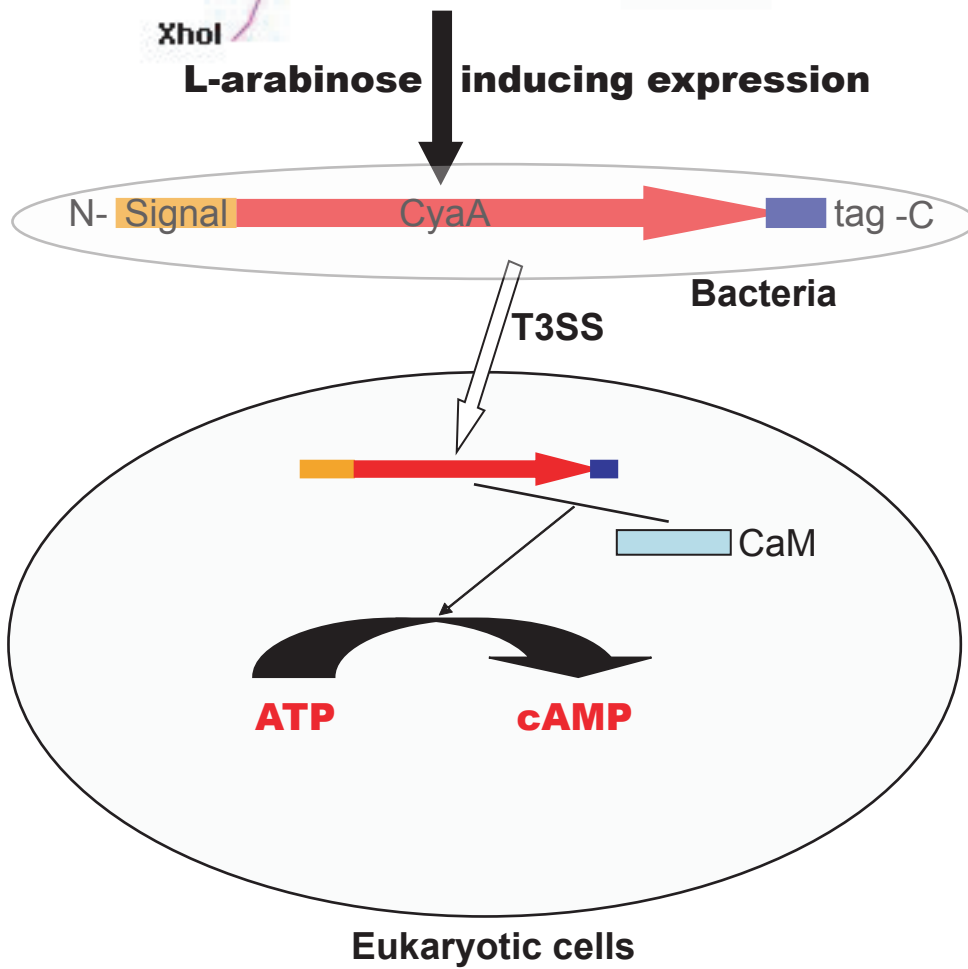

Supplement: Figure S4 — Principles of CyaA translocation assay. CyaA reporter plasmids inserted with N-terminal candidate signal sequences were transformed into bacteria of functional T3SSs. Under induction of L-arabinose, the mosaic protein fused with N-terminal candidate T3S signals, CyaA polypeptides, and C-terminal Myc-His double tags will be expressed. Under T3SS induction conditions, T3SS apparatus genes will be expressed and assembled. If the signal sequence cloned in reporter plasmid is true T3S signal, it will be specifically recognized by T3SS apparatus, and consequently the fusion protein will be translocated into contacting eukaryotic cells. In cytoplasm of eukaryotic cells, with the assistance of Calmodulin (CaM) protein, CyaA protein will exert its function to catalyze the reaction by which ATP is changed to cAMP. Therefore, the cAMP level will be increased significantly. (PDF) [file pone.0059754.s004.pdf]
