# Supplementary material for: Effective Identification of Bacterial Type III Secretion Signals Using Joint Element Features
Source: PLoS One. 2013 Apr 4;8(4):e59754. doi: 10.1371/journal.pone.0059754 (PMC3617162; doi:10.1371/journal.pone.0059754)
Supplement: Table S3 — Expression correlation with InvA and co-expression under InvA inducing conditions of newly identified Salmonella T3S proteins. (DOC) [file pone.0059754.s007.doc]

**Supplemental Table S3. Expression correlationship with InvA and co-expression under InvA inducing conditions of newly identified *Salmonella* T3S proteins**

| **Gene** | **PCC** | **SCC** | **Co-expression*** | **Category** |
| --- | --- | --- | --- | --- |
| yiiG | 0.6329 | 0.5603 | +++ | Newly Predicted and Validated |
| STM1791 | 0.6079 | 0.5020 | +++ | Newly Predicted and Validated |
| STM0281 | 0.7622 | 0.5965 | +++ | Newly Predicted and Validated |
| ygbI | 0.6366 | 0.4588 | +++ | Newly Predicted and validated |
| STM1870 | 0.9029 | 0.8019 | +++ | Newly Predicted and validated |
| STM2005 | 0.8020 | 0.6918 | +++ | Newly Predicted and validated |
| STM2486 | 0.1973 | 0.1375 | + | Newly Predicted and validated |
| ydiF | 0.8789 | 0.7191 | +++ | Newly Predicted and validated |

*Definition for co-expression under SPI-1 inducing conditions referred to Materials and Methods.
