# Supplementary material for: Effective Identification of Bacterial Type III Secretion Signals Using Joint Element Features
Source: PLoS One. 2013 Apr 4;8(4):e59754. doi: 10.1371/journal.pone.0059754 (PMC3617162; doi:10.1371/journal.pone.0059754)
Supplement: Table S5 — 3D structure predictions and comparison for T3S signal sequences. (DOC) [file pone.0059754.s009.doc]

**Table S5. 3D structure predictions and comparison for T3S signal sequences**

| **Property** | **T3S effectors** |
| --- | --- |
| Predicted structures with high confidence | *Shigella* IpaA, IpaD, IpaH1.4, IpgB1, IpgB2, VirA and MxiL; *Yersinia* YopE, YopH, YopO, YopP and YopR; *Chlamydophila* CopN and Q9Z8P7; *Chlamydia* Q3KMQ0 and CT621; *Salmonella* SlrP, SsaB, SseF, SptP and SopD; *EHEC* EspB; *Pseudomonas* AvrB, HopM1, HopAJ1, HopAN1, HopPtoA1Pma, Q9K2L5 and HopAI1; *Xanthomonas* XopD; *Rhizobium* NopL; *Burkholderia* BipB; *Bordetella* BopB; *Vibrio* VopC, VopF and VP1686; *Edwardsiella* EseC; *Citrobacter* EspZ; *Aeromonas* AexT; *Ralstonia* RSc1349 and RSc3401. |
| T3S signal cluster with similar structure | *Shigella* IpaA and IpaD; *Yersinia* YopP; *Chlamydophia* CopN; *Chlamydia* Q3KMQ0; *Salmonella* SlrP and SseF; *EHEC* EspB; *Pseudomonas* HopPtoA1Pma; *Xanthomonas* XopD; *Vibrio* VopF |
| T3S signal sequence pairs with highly similar structure | (1) *Yersinia* YopP, *EHEC* EspB and *Chlamydia* Q3KMQ0;  (2) *Pseudomonas* HopPtoA1Pma and *Xanthomonas* XopD;  (3) *Vibrio* VopF, *Rhizobium* NopL and *Shigella* IpgB1;  (4) *Vibrio* VopC and *Pseudomonas* HopAN1;  (5) *Ralstonia* RSc3401 and RSc1349. |
