# Supplementary material for: Effective Identification of Bacterial Type III Secretion Signals Using Joint Element Features
Source: PLoS One. 2013 Apr 4;8(4):e59754. doi: 10.1371/journal.pone.0059754 (PMC3617162; doi:10.1371/journal.pone.0059754)
Supplement: Table S6 — Plasmids used in this study. (DOC) [file pone.0059754.s010.doc]

**Supplemental Table S6. Plasmids used in this study**

| **Name** | **Origin** | **Property and usage** |
| --- | --- | --- |
| pMS107 | Gifted by Prof. Guy R Cornelis | Containing CyaA gene encoding sequence |
| pBADB-Myc-His | Ordered from Invitrogen | Amp+; Myc-His double tags; L-arabinose inducing expression |
| pBADB-CyaA-tag | Constructed in this study | pBADB-Myc-His plasmid inserted with CyaA encoding sequence |
| pBADB-sipC-CyaA-tag | Constructed in this study | pBADB-CyaA-tag inserted with sequence encoding N-terminal 100 aa of sipC gene at the 5' side of CyaA sequence. |
| pBADB-STM2005-CyaA-tag | Constructed in this study | pBADB-CyaA-tag inserted with sequence encoding N-terminal 100 aa of STM2005 gene at the 5' side of CyaA sequence. |
| pBADB-yiiG-CyaA-tag | Constructed in this study | pBADB-CyaA-tag inserted with sequence encoding N-terminal 100 aa of yiiG gene at the 5' side of CyaA sequence. |
| pBADB-yaaA-CyaA-tag | Constructed in this study | pBADB-CyaA-tag inserted with sequence encoding N-terminal 100 aa of yaaA gene at the 5' side of CyaA sequence. |
| pBADB-STM1791-CyaA-tag | Constructed in this study | pBADB-CyaA-tag inserted with sequence encoding N-terminal 100 aa of STM1791 gene at the 5' side of CyaA sequence. |
| pBADB-mdoH-CyaA-tag | Constructed in this study | pBADB-CyaA-tag inserted with sequence encoding N-terminal 100 aa of mdoH gene at the 5' side of CyaA sequence. |
| pBADB-STM1870-CyaA-tag | Constructed in this study | pBADB-CyaA-tag inserted with sequence encoding N-terminal 100 aa of STM1870 gene at the 5' side of CyaA sequence. |
| pBADB-STM2486-CyaA-tag | Constructed in this study | pBADB-CyaA-tag inserted with sequence encoding N-terminal 100 aa of STM2486 gene at the 5' side of CyaA sequence. |
| pBADB-ydiF-CyaA-tag | Constructed in this study | pBADB-CyaA-tag inserted with sequence encoding N-terminal 100 aa of ydiF gene at the 5' side of CyaA sequence. |
| pBADB-ygbI-CyaA-tag | Constructed in this study | pBADB-CyaA-tag inserted with sequence encoding N-terminal 100 aa of ygbI gene at the 5' side of CyaA sequence. |
| pBADB-STM0281-CyaA-tag | Constructed in this study | pBADB-CyaA-tag inserted with sequence encoding N-terminal 100 aa of STM0281 gene at the 5' side of CyaA sequence. |
| pBADB-Y1-CyaA-tag/ pBADB-Y2-CyaA-tag/ pBADB-Y3-CyaA-tag | Constructed in this study | pBADB-CyaA-tag inserted with sequence encoding N-terminal 100 aa of yeast gene DAA10219.1, DAA07267.1 or DAA10797.1 (Y1-Y3) at the 5' side of CyaA sequence. |
