# Supplementary material for: Effective Identification of Bacterial Type III Secretion Signals Using Joint Element Features
Source: PLoS One. 2013 Apr 4;8(4):e59754. doi: 10.1371/journal.pone.0059754 (PMC3617162; doi:10.1371/journal.pone.0059754)
Supplement: Table S7 — Primers used in this study. (DOC) [file pone.0059754.s011.doc]

**Supplemental Table S7. Primers used in this study**

| **Name** | **Sequence** | **Usage** |
| --- | --- | --- |
| cyaA-4 | 5'-GCGAATTCCAGCAATCGCATCAGG-3' | To amplify 5' 4-1215nt of cyaA gene |
| cyaA-1215 | 5'-GCTCTAGACTGTCATAGCCGGAAT-3' |
| pBADB-5' | 5'-ATG CCA TAG CAT TTT TAT CC-3' | To detect pBADB plasmid |
| pBADB-3' | 5'-GAT TTA ATC TGT ATC AGG-3' |
| sipC-F | 5'-GAGCTCGAGATTAATTAGTAATGTGGGAA-3' | To amplify 5' 4-300nt of sipC gene |
| sipC-R | 5'-CTGGAATTCAACCTCATTCGCTTTAGT-3' |
| 2005-F | 5'-GAGCTCGAGAGCATTAACCGATACCGCTG-3' | To amplify 5' 4-300nt of STM2005 gene |
| 2005-R | 5'-CTGGAATTCCTTCTGCCATGACTCCGCT-3' |
| yiiG-F | 5'-GAGCTCGAGAACCCTGGGCGCAACAGGA-3' | To amplify 5' 4-300nt of yiiG gene |
| yiiG-R | 5'-CTGGAATTCCGCGTAACGCGCCAGACT-3' |
| yaaA-F | 5'-GAGCTCGAGACTGATTCTGATTTCACCTGC-3' | To amplify 5' 4-300nt of yaaA gene |
| yaaA-R | 5'-CTGGAATTCAAAATCCGCGTCGTTGAACGT-3' |
| 1791-F | 5'-GAGCTCGAGAAGCAACGCCTTTTTTCATCTG-3' | To amplify 5' 4-300nt of STM1791 gene |
| 1791-R | 5'-CTGGAATTCATGCTGAATGCGTACCGAGA-3' |
| mdoH-F | 5'-GAGCTCGAGAAATAAAACAACTGAGTATATTGACG-3' | To amplify 5' 4-300nt of mdoH gene |
| mdoH-R | 5'-CTGGAATTCACGGCCAACCGGGTTGGTTC-3' |
| 1870-F | 5'-GAGCTCGAGAGAACAGGACACAACTGAACA-3' | To amplify 5' 4-300nt of STM1870 gene |
| 1870-R | 5'-CTGGAATTCCTCCGGCTCTGCTGCTGGT-3' |
| 2486-F | 5'-GAGCTCGAGACGCTGGCAAGGGCGTCGTG-3' | To amplify 5' 4-300nt of STM2486 gene |
| 2486-R | 5'-CTGGAATTCCATTTTTTGAAAGAGTTGACCCCAG-3' |
| ydiF-F | 5'-GAGCTCGAGAAAAATCAATAAGCCATCAC-3' | To amplify 5' 4-300nt of ydiF gene |
| ydiF-R | 5'-CTGGAATTCACGGGGAGATTGCCCCCAA-3' |
| ygbI-F | 5'-GAGCTCGAGAATACCTACTGAGCGTCGAC-3' | To amplify 5' 4-300nt of ygbI gene |
| ygbI-R | 5'-CTGGAATTCACCTGCATCAAGGTAGATAC-3' |
| 0281-F | 5'-GAGCTCGAGAAGCTGGAATGACCGCGTAG-3' | To amplify 5' 4-300nt of STM0281 gene |
| 0281-R | 5'-CTGGAATTCCGCCAGACAAATCTGCTGG-3' |
| Y1-F | 5'-GAGCTCGAGAGGTTCATCTGGTTCAAAAT-3' | To amplify 5' 4-300nt of DAA10219.1 gene |
| Y1-R | 5'-CTGGAATTCAGAAATATCGGATGATGTGT-3' |
| Y2-F | 5'-GAGCTCGAGAAGTTTTAGACATTTCAAGAG-3' | To amplify 5' 4-300nt of DAA07267.1 gene |
| Y2-R | 5'-CTGGAATTCTAATGGCCTCGGTAAAGGT-3' |
| Y3-F | 5'-GAGCTCGAGAATGCGTGGTTTCAAGCAAAG-3' | To amplify 5' 4-300nt of DAA10797.1 gene |
| Y3-R | 5'-CTGGAATTCGCTGCTGGAAGAAGGGGTCG-3' |
